# Grinding Metamorphic Layer of Bearing Steel: Formation Mechanisms, Characterization, and Process Parameter Effects

**DOI:** 10.3390/ma19153334

**Published:** 2026-08-05

**Authors:** Jiayu Guo, Tao Xia, Dingbo Cao, Xue Liu, Wei Zhang, Yong Liu, Jingchuan Zhu

**Affiliations:** School of Materials Science and Engineering, Harbin Institute of Technology, Harbin 150001, China

**Keywords:** grinding metamorphic layer, bearing steel, gradient structure, microstructural characterization, formation mechanism

## Abstract

Grinding is the final precision machining step for bearing rings, which induces subsurface gradients in microstructure and mechanical properties. Rolling contact fatigue life and service reliability are directly determined by the gradients. Current research of the grinding metamorphic layer in bearing steels is synthesized in this review. The formation mechanisms, characterization approaches, and the influence of grinding parameters on metamorphic layers is covered. The coupled thermal–mechanical–phase transformation framework encompasses heat-driven phase transformation, high-strain-rate gradient plastic deformation, and their interactions, which collectively govern the formation of the three-layer gradient structure. When the surface temperature exceeds the austenitization threshold, the governing regime shifts from mechanically dominated to thermally dominated, producing an abrupt increase in white layer thickness and concurrent dark layer softening. The capabilities and limitations of characterization techniques for probing the gradient microstructure and residual stress profile are evaluated. The influence of grinding depth, wheel speed, feed rate, wheel characteristics, and cooling conditions on the metamorphic layer is analyzed. The areas requiring deeper investigation are identified. These insights aim to establish correlations between the grinding process and the surface integrity and service performance of bearing components, and to provide directions for future research on the grinding metamorphic layer.

## 1. Introduction

Rolling bearings are fundamental components of rotating machinery, serving critical roles in high-end equipment such as aircraft engines, wind turbines, and precision machine tools [1,2]. The surface integrity and service reliability of bearing rings directly govern the operational accuracy and service life of these systems [3,4,5]. The complete manufacturing chain for bearing rings includes forging, spheroidizing annealing, turning, heat treatment, grinding, and superfinishing, which culminates in grinding as the final precision machining operation and determines the dimensional accuracy, surface topography, and subsurface state of the bearing raceway [6,7]. Unlike other material removal processes, grinding relies on high-speed friction and cutting between randomly distributed abrasive grains and the workpiece surface, generating extreme local temperatures and strain rates that drive intense thermal–mechanical coupling. This coupling fundamentally alters the subsurface microstructure and properties of the material [8,9].

This grinding-induced subsurface variation, termed the metamorphic layer, refers to a gradient-structured region beneath the machined surface in which the microstructure, phase composition, mechanical properties, and residual stress state deviate substantially from those of the bulk material [10,11]. The metamorphic layer typically exhibits a multilayer architecture. The topmost layer, known as the white layer, consists of nanocrystalline martensite and carbon-supersaturated retained austenite. Beneath it lies the dark layer, a region of over-tempered martensite decorated with fine carbide precipitates. Further below, a transition zone gradually bridges to the unaffected bulk substrate [12,13]. The formation of these sublayers is governed by the coupled and competing effects of phase transformation and severe plastic deformation, driven by grinding-induced thermal and mechanical loads [12,14,15]. The thickness, phase constitution, hardness gradient, and residual stress profile of the metamorphic layer are directly determined by the grinding process parameters and the initial metallurgical state of the bearing steel [16,17].

Over the past two decades, substantial progress has been made toward understanding the formation mechanisms of grinding metamorphic layers in hardened steels, particularly in AISI 52100 and 8Cr4Mo4V bearing steels [10,18,19]. Experimental studies have systematically examined the influence of grinding parameters on the thickness, hardness, and residual stress of the metamorphic layer [6,7,20]. A range of characterization techniques, including optical microscopy, scanning and transmission electron microscopy, electron backscatter diffraction, X-ray diffraction, and Barkhausen noise analysis, have been employed to probe the microstructural and mechanical property gradients within the metamorphic layer [19,21,22]. On the simulation front, finite element methods have been applied to predict temperature fields, phase transformation kinetics, and residual stress formation [16,23]. The effects of the metamorphic layer on rolling contact fatigue, wear behavior, and corrosion resistance have also been investigated on bearing components [24,25,26,27]. Moreover, comparative studies between grinding and alternative finishing processes have highlighted the unique surface modification characteristics imparted by grinding [6,15,28].

Several existing reviews have addressed aspects of this topic but with distinct limitations. Bhadeshia [29] provided a comprehensive overview of bearing steel metallurgy, yet did not address grinding-induced surface modification. El Laithy et al. [3] reviewed rolling contact fatigue mechanisms in bearings, but without considering the grinding-induced surface state as a precursor. Umbrello et al. [14] modeled white and dark layer formation in hard machining, which focuses on hard turning rather than grinding and on modeling rather than the full characterization-to-performance chain. Reviews on grinding burn detection [30,31] are confined to non-destructive evaluation techniques. None of these existing reviews provide a systematic synthesis that specifically targets the grinding-affected layer in bearing steel across the full chain from physical formation mechanisms to microstructural characterization, process parameter control, and service performance implications.

**Figure 1:**
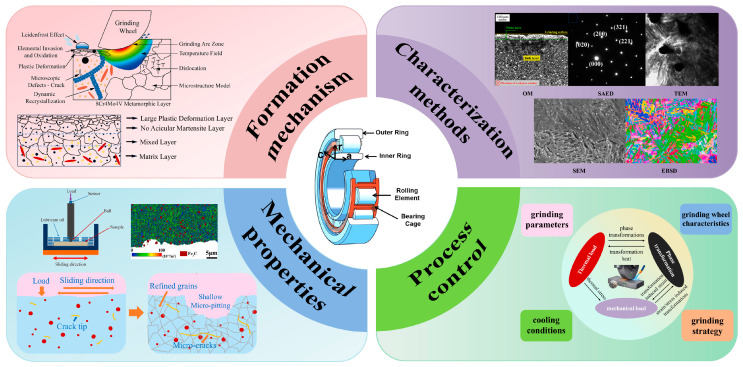
Overview of existing research on the metamorphic layer in bearing rings, systematically illustrating the formation mechanisms, characterization methods, process control strategies, and their roles in governing component mechanical properties.

Therefore, as shown in Figure 1, this review aims to analyze the current research results of the grinding metamorphic layer in bearing rings, specifically the formation process of the metamorphic layer under thermal-driven, mechanically driven, and coupled thermal–mechanical pathways. The capability of various characterization techniques for resolving the gradient microstructure and mechanical properties was assessed. The influence of grinding process parameters on metamorphic layer characteristics and the associated surface integrity control strategies was reviewed.

## 2. Fundamentals of Bearing Steel Grinding

The metamorphic layer is a material response of bearing steel to extreme thermal–mechanical coupling. Its formation and evolution are fundamentally governed by two factors: the metallurgical characteristics of the workpiece material and the thermal–mechanical coupling features of the grinding process. Therefore, before delving into the formation mechanisms, characterization methods, and performance implications of the metamorphic layer, it is necessary to clarify the metallurgical characteristics and grinding response of bearing steel, the thermal–mechanical coupling mechanisms in the grinding process, and the definition and structural framework of the grinding metamorphic layer.

### 2.1. Development and Microstructural Characteristics of Bearing Steel

The initial microstructure of bearing steel determines both the thermodynamic driving force for phase transformation during grinding and the kinetic pathway of carbon redistribution. Differences in carbide type, retained austenite content, and martensitic tempering resistance across bearing steel grades create distinct initial conditions, leading to markedly different metamorphic layer structures and residual stress states under identical grinding parameters [18,19].

Carbide type is the most critical microstructural feature distinguishing the grinding response of different bearing steels. As shown in Table 1, AISI 52100 and M50 steels differ fundamentally in their carbide species. AISI 52100 is rich in M_7_C_3_ carbides, which have relatively low thermal stability. When the grinding depth exceeds 25 μm and the surface temperature rises above 778 °C, fine nanoscale carbides in the superficial layer dissolve rapidly within millisecond-scale thermal cycles due to their extremely short diffusion distances. Carbon and alloying elements redissolve into the austenite matrix, providing the primary carbon source for phase transformation of white layer formation. In contrast, submicron- to micron-scale secondary carbides undergo only partial dissolution and refinement owing to the slow diffusion of substitutional alloying elements and the extremely short contact time [10,32]. By comparison, MC-type carbides in M50 steel exhibit substantially higher thermal stability. Studies on the high-temperature decomposition behavior of M_2_C primary carbides show that they progressively decompose into M_6_C and MC at elevated temperatures [33]. However, their decomposition temperature far exceeds the dissolution temperature of M_7_C_3_ in AISI 52100, so only minor partial dissolution occurs under equivalent grinding thermal cycles [34,35]. The survival of abundant undissolved carbides in the white layer shifts its formation mechanism from purely driven by phase-transformation to thermal–mechanical coupling. Moreover, the retention of MC carbides fundamentally alters the sublayer architecture of the metamorphic layer. It blurs the boundary between the white layer and the dark layer, producing broader, more continuous gradient transitions among sublayers. Zhang further proposed a gradient zoning method for such high-alloy steels, dividing the metamorphic layer into a severe plastic deformation zone and an acicular-martensite-free zone based on deformation degree [19]. Liu et al. revealed through Gleeble physical simulation that grinding-induced thermal–mechanical coupling can lower the $A_{c1}$ temperature by 50–80 °C, thereby triggering austenitization at lower grinding temperatures [18].

Retained austenite (RA) content and its decomposition kinetics constitute the second critical factor influencing residual stress in the metamorphic layer. The typical RA content is 5–15% in AISI 52100 steel and 15–25% in M50 steel due to its higher alloy content. Torsional fatigue studies on carburized AISI 8620 steel indicate that RA transformation to martensite under cyclic loading is accompanied by approximately 5–8% volume expansion, which serves as the primary microstructural mechanism for generating compressive residual stress [36]. However, an RA transformation amount exceeding 10% is required to produce effective residual stress, and a Hertzian contact stress of approximately 5 GPa can rapidly exhaust the transformation potential of RA. Furthermore, the initial heat treatment state of the bearing ring forms the microstructural basis for metamorphic layer formation during grinding. Using Abaqus finite element simulation, Wang found that M50 steel rings subjected to high-pressure nitrogen gas quenching exhibit approximately 810 MPa of compressive residual stress, while water quenching can reach 1436 MPa. The compressive stress also shows a pronounced size effect: 550 MPa for a 30 mm diameter and 1030 MPa for 50 mm. This initial stress field is directly superimposed onto the subsequent grinding-induced stress [37]. Cryogenic treatment can further regulate the initial RA content and stress state: treatment at −80 °C for 1.5 h effectively reduces the residual stress on the raceway surface and improves ring roundness [38]. Using neutron diffraction to measure through-thickness residual stress across the entire manufacturing sequence of thin-walled bearing rings, Bedekar revealed a cumulative evolution pattern in which subsurface stress gradually shifts from tensile to compressive, while the core stress remains unchanged throughout [23]. These results indicate that the initial heat treatment state prior to grinding exerts an influence on the final stress state and metamorphic layer structure comparable to that of the grinding process itself.

**Table 1:** Comparison of basic material property parameters of various bearing steels.

Steel Grade	Carbide Type	Hardness	Service Temperature	References
AISI 52100	M_7_C_3_	58–64 HRC	~150 °C	Bhadeshia (2012) [29]; E.V. Zaretsky (2012) [39]
100Cr6	M_7_C_3_	58–64 HRC	~150 °C	Yin et al. (2022) [40]; Sidoroff et al. (2014) [41]
GCr15SiMn	M_7_C_3_	58–62 HRC	~150 °C	Xu et al. (2006) [42]
AISI 440C	M_23_C_6_ + M_7_C_3_	58–60 HRC	~150 °C	R. Rejith (2024) [43] G. Prieto (2020) [44]
M50	MC + M_2_C	60–65 HRC	~315 °C	Bhadeshia (2012) [29]; Miao et al. (2026) [45]
M50 NiL	MC + M_2_C	Surface ~60 HRC; core 40–45 HRC	~315 °C	C. Anil Kumar Reddy (2022) [46]; B. Venkatesh (2021) [47]

### 2.2. Establishment of a Grinding Thermal–Mechanical Model

The following subsections present the fundamental equations that govern the grinding thermal–mechanical response. These models are not intended as a standalone numerical framework, but rather provide the quantitative vocabulary, such as heat flux, strain rate, phase transformation kinetics, and residual stress, which underpins the mechanistic discussion of experimental observations in Section 3, Section 4 and Section 5.

#### 2.2.1. Grinding Force of a Single Abrasive Grain

**Figure 2:**
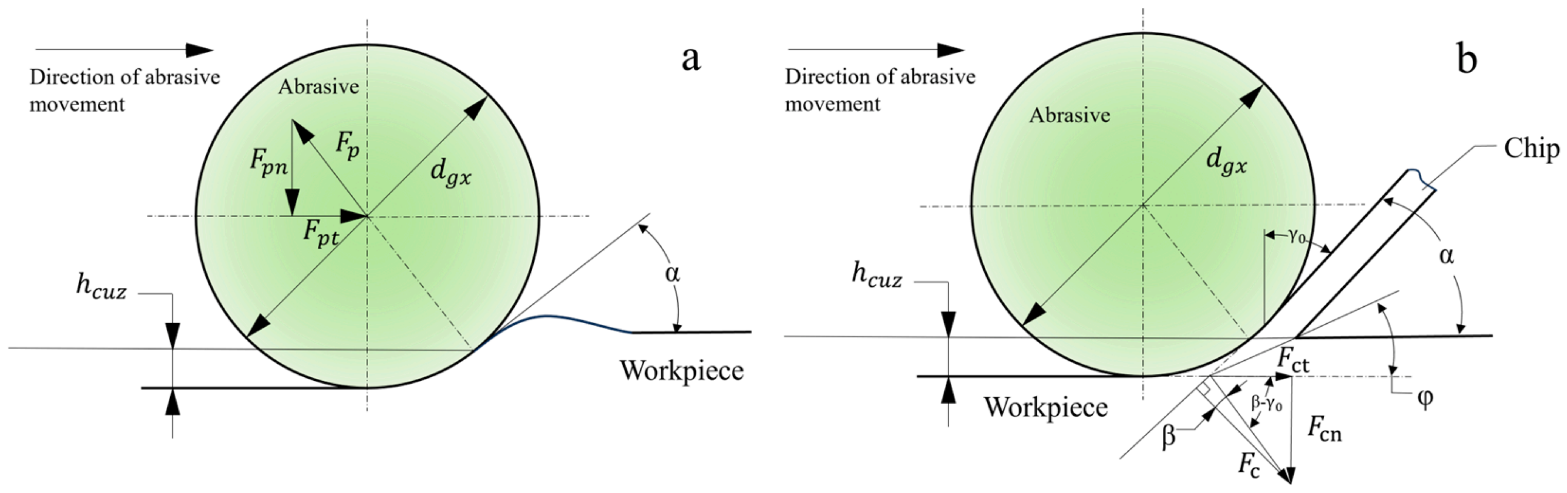
Force analysis of a single abrasive grain [48]. (**a**) Force diagram of a single abrasive plowing the workpiece, where $F_{p}$ is the grinding force of the abrasive grain, $F_{pn}$ is the normal grinding force, $F_{pt}$ is the tangential plowing force, $d_{gx}$ is the abrasive grain diameter, α is the cutting angle, and $h_{cuz}$ is the penetration depth; (**b**) force diagram of a single abrasive cutting the workpiece, where $F_{c}$ is the abrasive cutting force, $F_{cn}$ is the normal cutting force, $F_{ct}$ is the tangential cutting force, $\gamma_{0}$ is the rake angle, φ is the shear angle, and β is the friction angle.

The force analysis of a single abrasive grain plowing the workpiece is shown in Figure 2a.

Hecker, using the definition and derivation method of Brinell hardness, derived the grinding force for a single abrasive grain as [49]

(1) $$H=\frac{2F}{\pi D\left(D-\sqrt{D^{2}-b^{2}}\right)},$$

where F is the pressure, D is the diameter of the carbide ball, and b is the groove diameter. Hecker considered that the plowing process of abrasive grains is analogous to Brinell hardness testing, in which intense plastic flow occurs in the workpiece material. Therefore, the carbide ball diameter D can be replaced by the abrasive grain diameter $d_{gx}$, and the expression $\left(D-\sqrt{D^{2}-b^{2}}\right)/2$ denotes the penetration depth $h_{cuz}$. Substituting the carbide ball diameter D in Equation (1) with the abrasive diameter $d_{gx}$, Hecker derived the grinding force for a single abrasive grain $F_{p}$:

(2) $$F_{p}=\pi Hd_{gx}h_{cuz},$$

Chen et al. further derived the equations for the normal force ${\;F}_{pn}$ and tangential force $F_{pt}$ [50]:

(3) $$\left\{\begin{array}{c} F_{pt}=\pi Hd_{gx}h_{cuz}(sin\alpha +\mu_{d}cos\alpha )\\ {\;F}_{pn}=\pi Hd_{gx}h_{cuz}(cos\alpha -\mu_{d}sin\alpha ) \end{array}\right.,$$

where *μ* is the friction coefficient between the workpiece and the abrasive grain.

The cutting angle α can be expressed as

(4) $$\alpha =arccos\left(1-\frac{2h_{cuz}}{d_{gx}}\right),$$

Equations (2) and (3) are applicable only to the plowing stage. For abrasive grains in the cutting stage, a new cutting force model must be established. The force analysis of a single abrasive grain cutting the workpiece is shown in Figure 2b, where $F_{c}$ is the abrasive cutting force, $F_{cn}$ is the normal cutting force, $F_{ct}$ is the tangential cutting force, $\gamma_{0}$ is the rake angle, φ is the shear angle, and β is the friction angle.

Jiang et al. established the expression for the single abrasive cutting force in the cutting stage [51]:

(5) $$\left.\left\{\begin{array}{c} F_{ct}=\tau_{s}\frac{h_{cuz}d_{gx}}{4}{\left(\frac{\cos\gamma_{0}}{sin\varphi \;cos(\varphi -\gamma_{0})}\right)}^{n}\frac{cos(\beta -\gamma_{0})}{sin\varphi \;cos(\varphi +\beta -\gamma_{0})}\\ F_{cn}=\pi \tau_{s}\frac{h_{cuz}d_{gx}}{4}{\left(\frac{\cos\gamma_{0}}{sin\varphi \;cos(\varphi -\gamma_{0})}\right)}^{n}\frac{sin(\beta -\gamma_{0})}{sin\varphi \;cos(\varphi +\beta -\gamma_{0})} \end{array}\right.\right.,$$

where $\tau_{s}$ is the shear yield strength of the material, and n is the strengthening coefficient.

The rake angle $\gamma_{0}$, shear angle φ, and friction angle β can be expressed as

(6) $$\left.\gamma_{0}=-\arcsin\left(1-\frac{2h_{cuz}}{d_{gx}}\right)\right.,$$

(7) $$\varphi =\frac{\pi }{4}-\frac{\beta }{2}+\frac{\gamma_{0}}{2},$$

(8) $$\beta =arctan{\;\mu }_{g},$$

where $\mu_{g}$ is the friction coefficient between the abrasive grain and the workpiece.

#### 2.2.2. Grinding Heat Flux Density

Most of the grinding heat is transferred to the workpiece within the grinding arc zone. Therefore, the grinding contact arc length is a critical parameter for calculating the heat flux density and can be expressed as

(9) $$l_{h}=\sqrt{a_{s}d_{wh}},$$

where $d_{wh}$ is the grinding wheel diameter, $a_{s}$ is the grinding depth, $a_{s}=1/2v_{f}\left(60/n_{w}\right)$, $v_{f}$ is the workpiece feed rate, and $n_{w}$ is the speed of workpiece rotation.

Based on the tangential component of the grinding force, the total heat source in the grinding arc can be expressed as

(10) $$q_{l}=\frac{F_{tr}v_{s}}{b_{p}l_{h}},$$

where $F_{tr}$ is the tangential component of the grinding force,$\;v_{s}$ is the grinding wheel speed, $b_{p}$ is the grinding contact width, and $b_{p}l_{h}$ is the instantaneous contact area between the wheel and the workpiece.

The heat partition ratio between the grinding wheel and the workpiece is

(11) $$\varepsilon_{ws}=\frac{1}{1+\frac{0.97k_{g}}{\sqrt{r_{0}}v_{s}{\left(k\rho c\right)}_{w}}},$$

where $k_{g}$ is the thermal conductivity of the abrasive grain; $k_{w}$, $\rho_{w}$, and $c_{w}$ are the thermal conductivity, density and specific heat capacity of the workpiece material, respectively; and $r_{0}$ is the contact radius of the abrasive grain.

The thermal diffusivity of the workpiece $\alpha_{w}$ can be expressed as

(12) $$\alpha_{w}=\frac{k_{w}}{\rho_{w}c_{w}},$$

Wang et al. calculated the moving heat source element by integrating the contact length [52]. Grinding temperature is typically analyzed using moving heat source theory, with the workpiece modeled as a semi-infinite solid. Compared with the heat source on the contact surface, the heat source on the finished surface is more convenient for grinding temperature field analysis. The heat source profile on the finished surface (PHSF) is modeled with different shapes depending on grinding conditions, such as rectangular, triangular, and parabolic heat flux distributions.

The PHSF shape depends on the contact angle φ and the Péclet number $P_{e}$. The contact angle φ is calculated by $sin\varphi =a_{p}/l_{h}$, and the Péclet number reflects the influence of the contact angle on the simulated temperature field; the smaller the Péclet number, the smaller the influence of the contact angle. The equation is

(13) $$P_{e}=\frac{v_{w}l_{h}}{{4\alpha }_{w}},$$

where $v_{w}$ is the workpiece velocity.

**Figure 3:**
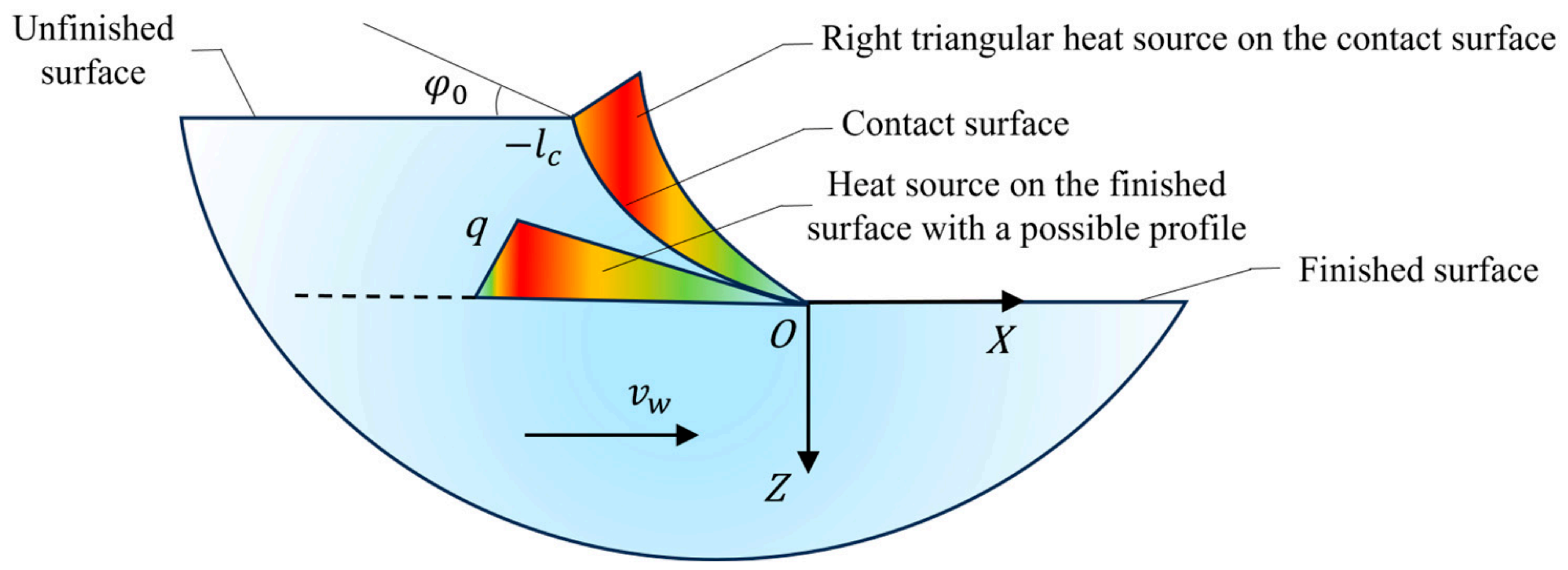
Heat flux distribution on the contact surface and the finished workpiece surface [53].

Wang et al. [52] concluded that when the contact angle is less than 5°, the PHSF is modeled as rectangular; when the contact angle ranges from 5° to 10° and Pe < 5, the PHSF is triangular; when the contact angle exceeds 10° and Pe ≥ 5, the PHSF is parabolic. In their study, all contact angles calculated under the given grinding conditions were less than 5°. Therefore, the PHSF was assumed to be right-triangular. Figure 3 shows the heat flux distributions on the contact surface and the finished surface.

The heat flux density transferred to the workpiece is

(14) $$q_{wl}=\frac{\varepsilon_{ws}\left(q_{l}-q_{chl}\right)}{1+\varepsilon_{ws}h_{f}/h_{w}},$$

where $q_{l}$ is the total heat source in the grinding arc and $q_{chl}$ is the heat carried away by chips. Based on the abrasive grain trajectory, chip formation during grinding is minimal; thus, $q_{chl}$ = 0. $h_{f}$ is the convective heat transfer coefficient of the grinding coolant, and $h_{w}$ is the heat transfer coefficient of the workpiece, given by

(15) $$h_{w}=\frac{3}{2C}\sqrt{\frac{k_{w}\rho_{w}c_{w}v_{w}}{l_{h}}},$$

where the parameter C is related to the value of $P_{e}$ as follows:

(16) $$C=\left\{\;\begin{array}{c} 1.0\;\;\;\;\;\;\;\;\;\;\;\;\;\;\;\;\;\;\;\;\;\;\;\;\;\;\;\;\;\;\;\;\;\;\;\;\;P_{e}>10\\ \frac{0.95}{\pi }\sqrt{2\pi +P_{e}/2}\;\;\;\;\;\;\;\;\;\;\;\;\;0.2\leq P_{e}\leq 10\\ 0.76\;\;\;\;\;\;\;\;\;\;\;\;\;\;\;\;\;\;\;\;\;\;\;\;\;\;\;\;\;\;\;\;\;\;\;P_{e}<0.2 \end{array}\right.,$$

### 2.3. Thermal–Mechanical Analysis of the Grinding Process

The temperature field analysis of the grinding contact zone provides the physical basis for understanding the phase transformation driving mechanisms of the metamorphic layer [54]. Based on Jaeger’s moving heat source theory, Chang equated the heat input in the grinding zone to a rectangular heat source moving along the workpiece surface at the feed rate [8]. When grinding with Al_2_O_3_ wheels, approximately 80% of the total energy is transferred into the workpiece, with only about 20% going into the grinding wheel abrasives, while the energy carried away by chips is negligible. Under typical conditions, the peak surface temperature can reach 1000 °C but drops to approximately 400 °C at a depth of only 100 μm, establishing an extreme temperature gradient in the depth direction. Alagumurthi et al. systematically measured the energy partition ratio in cylindrical grinding of AISI 3310 steel, obtaining a theoretical value of Rth = 0.92, a grit contact length of 0.105 mm, a contact time of 4.02 μs, and a heat flux into the workpiece of Qw = 278 W/mm^2^ [9]. Using the Taguchi method to optimize grinding parameters, they found that the importance ranking of parameters affecting heat generation was depth of cut > workpiece speed > wheel speed > number of passes, and that the optimal parameter combination significantly reduced thermal damage. For micro-grinding temperature prediction, Zhao introduced a Taylor factor model to quantify the effect of crystallographic orientation in multiphase materials on grinding energy, while also accounting for the latent heat of phase transformation and the Hall–Petch grain size strengthening effect [53]. Furthermore, the anisotropy of thermal conductivity caused by crystallographic orientation can lead to peak temperature differences of 100–200 °C under identical grinding conditions, and the latent heat released during phase transformation raises the temperature by an additional 50–80 °C. Micro-grinding simulations show peak temperatures in the range of 600–900 °C, with a temperature drop of approximately 50% within the first 30 μm of depth.

From a mechanical perspective, the negative rake angle cutting geometry of abrasive grains generates extreme strain rates during grinding. Yao developed a modified Johnson–Cook constitutive model incorporating grain size effects to predict grinding forces in 40Cr steel. They found that when both dynamic recrystallization (DRX) and phase transformation occur simultaneously, the flow stress decreases by approximately 15–20%, and the modified model improves prediction accuracy by about 12% compared with the standard Johnson–Cook model [55]. In high-strain-rate grinding experiments, the specific grinding energy was measured at 60–120 J/mm^3^, and the normal grinding force consistently remained 2–3 times the tangential force, reflecting the compression-dominated characteristics of negative-rake-angle abrasive grains [56]. Within the superficial region (depth < 5 μm), complete reverse austenite transformation and the formation of nanocrystals (30–100 nm) were observed even at relatively low temperatures, indicating that high strain rates alone are sufficient to drive surface microstructural evolution. The mechanism lies in the sharp increase in dislocation density within millisecond time scales, providing sufficient driving force for dynamic recrystallization. Poulachon further confirmed that in the formation of the white layer during hard turning, thermal mechanisms dominate in the fully developed white layer region, while mechanical mechanisms contribute significantly only in the subsurface transition zone, with higher cutting speeds producing thicker white layers [57]. Cheng compared the responses of three initial microstructures, finding that the quenched condition produced a surface white layer 5–8 μm thick with high hardness [58]. The tempered condition yielded a white layer of only 2–4 μm, and the annealed condition produced the thickest white layer but with the most severe surface damage. The surface compressive residual stress was −400 to −600 MPa for the quenched condition but only −200 to −300 MPa for the tempered condition, directly confirming the controlling role of initial microstructure on the mechanical response.

The grinding contact zone can be characterized by depth-dependent extreme conditions. The surface layer simultaneously experiences transient temperatures exceeding the phase transformation point and contact stresses 2–3 times the yield strength. The subsurface layer is subjected only to tempering-range thermal exposure, with mechanical input substantially attenuated. At greater depths, both thermal and mechanical effects approach zero. This depth-dependent gradient directly produces the three-layer gradient metamorphic structure comprising the white layer, dark layer, and transition zone [57,59,60]. Based on moving heat source theory, Jun established an analytical model for the thermally metamorphic layer depth in gear form grinding, predicting that the metamorphic layer depth increases with grinding depth and decreases with wheel speed, ranging from tens to hundreds of micrometers, which is consistent with the temperature gradient of 1000 °C at the surface and approximately 400 °C at 100 μm of depth [59]. Using a coupled FEM-CA model, Guo further elucidated the microstructural mechanism of the hardness gradient [60]: the surface layer, with temperature exceeding $A_{c3}$, undergoes complete austenitization followed by rapid quenching, forming fine martensite (high hardness); the subsurface layer, with temperature between $A_{c1}$ and $A_{c3}$, experiences partial austenitization, forming a mixed microstructure (moderate hardness); and deeper regions, with temperature below $A_{c1}$, undergo no phase transformation (base hardness). This establishes the correspondence where the white layer corresponds to the surface region above $A_{c1}$ that undergoes austenitization and re-quenching, the dark layer corresponds to the subsurface region within the tempering temperature range where over-tempering precipitation occurs, and the transition zone corresponds to the deeper region where plastic deformation gradually attenuates.

## 3. Formation Mechanisms of the Grinding Metamorphic Layer

### 3.1. Sublayer Evolution Driven by Phase Transformation

Phase transformation driven by grinding heat is the primary mechanism underlying the formation of the sublayer architecture in the metamorphic layer. Unlike the near-equilibrium heating conditions of conventional heat treatment, the grinding contact zone experiences extreme non-equilibrium thermal cycles on a millisecond timescale. In situ temperature measurements indicate that when hardened AISI 52100 steel is dry-ground with a CBN wheel, the instantaneous peak surface temperature can reach 1132 °C, with a heating rate as high as 1.3 × 10^5^ °C/s. Upon wheel disengagement, the surface undergoes rapid self-quenching at approximately 2.1 × 10^3^ °C/s [61]. Under high-speed grinding conditions, the heating and cooling rates can further increase to 10^5^–10^6^ °C/s and >10^4^ °C/s, respectively [62]. This intense heat flow establishes a steep temperature gradient from the workpiece surface inward, subjecting different depth intervals to distinct peak temperatures ranging from the tempering range through the austenitization threshold up to the melting point, thereby triggering disparate phase transformation pathways [8,53,63]. It is important to note that under such far-from-equilibrium rapid heating conditions, the drastically shortened diffusion time causes the austenitization start temperature to shift upward with increasing heating rate, and the corresponding phase transformation kinetics transitions from diffusion-controlled to interface-controlled [64]. This non-equilibrium characteristic determines whether reverse austenite transformation can be initiated in the surface layer and, consequently, the distribution of phase transformation pathways across sublayers.

Once the peak surface temperature exceeds the shifted austenitization temperature during the rapid heating phase of grinding, reverse transformation from martensite to austenite occurs [10,61]. This process is accompanied by carbide dissolution and refinement: nanoscale carbides dissolve rapidly within the extremely short timeframe due to their fine size, serving as the primary source for the recovery of retained austenite content in the surface layer, whereas micron-scale secondary carbides, limited by the extremely short contact time, undergo only partial dissolution with their carbon entering the austenite lattice [10,32]. Subsequent rapid self-quenching drives the carbon-enriched austenite below the Ms temperature, forming high-carbon martensite [32,61]. The supersaturated carbon occupying interstitial sites in α-Fe induces asymmetric lattice distortion. The elevated distortion energy predisposes the martensitic transformation to proceed via a twinning mechanism, ultimately producing a hardened white layer composed of nanocrystalline martensite [61]. The grain size within this white layer can be refined to several tens of nanometers, while the retained austenite content rebounds from approximately 4.5% in the base material to 19–33% [32,62], which is a signature of reaustenitization and rapid quenching-dominated phase transformation.

**Figure 4:**
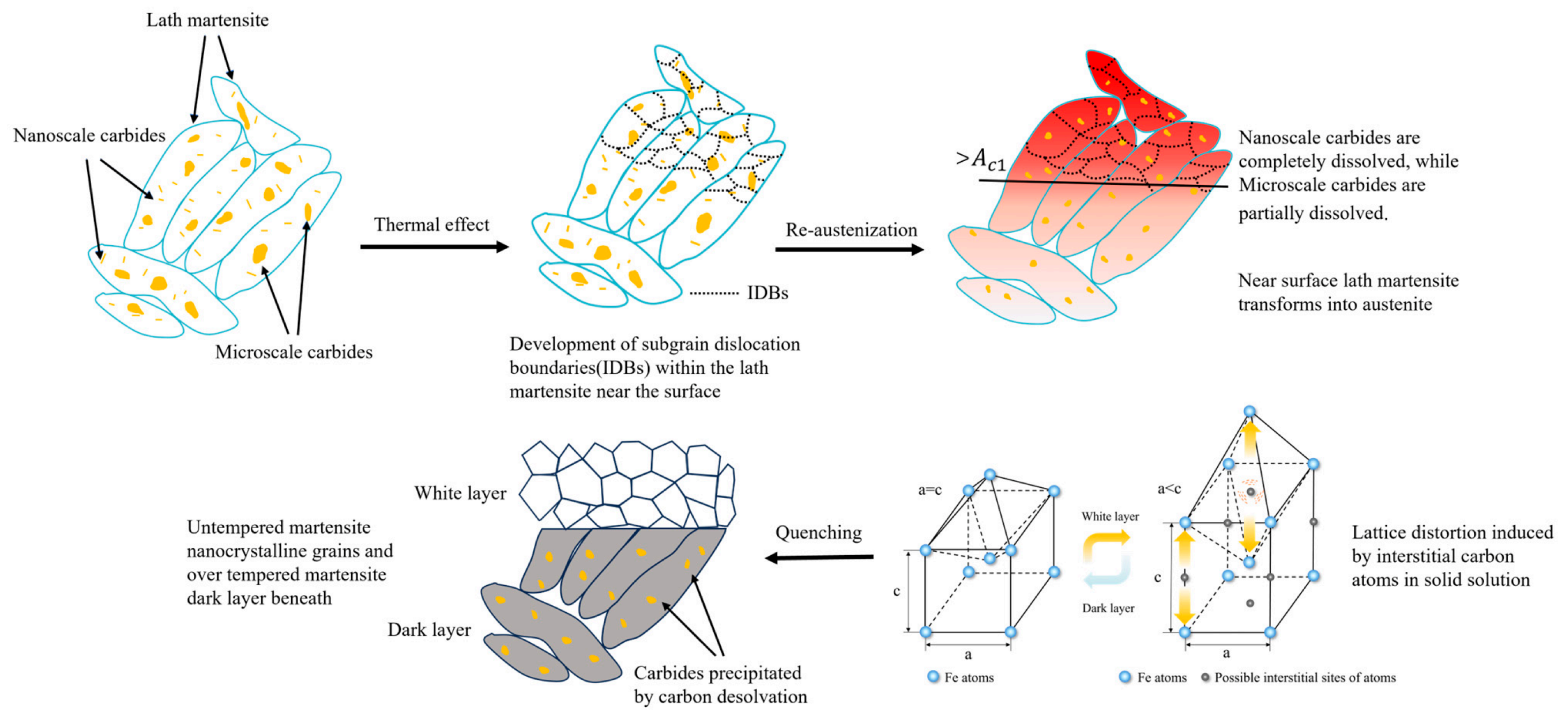
Schematic illustration of the microstructural evolution of the white layer and dark layer induced by grinding-heat-driven phase transformation.

In contrast to the fully austenitized surface region, the subsurface zone at depths exceeding approximately 10–20 μm experiences peak temperatures between the tempering range and the austenitization temperature. Within this temperature window, supersaturated carbon precipitates from the α-Fe lattice, forming transitional carbides that progressively coarsen. The lattice tetragonality c/a returns to unity, producing a softened dark layer consisting of over-tempered martensite and ferrite [14,15,61]. As the peak temperature increases and the high-temperature dwell time extends, carbides grow and coarsen while the matrix carbon content diminishes, creating a distinct carbon-depleted zone [61]. This depth-direction carbon redistribution is directly evidenced by compositional measurements: at grinding depths of 25–40 μm, the mean carbon concentration is approximately 1.15–1.25% in the white layer and 0.96–1.0% in the dark layer—intermediate between the white layer and the base material—with a dark layer thickness reaching 20–40 μm [10,14]. A transition zone of continuously varying carbon concentration exists between the white layer and the dark layer, whose thickness is closely related to the thermal penetration depth and carbon diffusion distance. Taken together, the two competing phase transformation pathways—surface austenitization followed by quenching on one hand, and subsurface tempering decomposition on the other—jointly govern the competitive formation and gradient distribution of the white and dark layers. Figure 4 illustrates the microstructural evolution of the white layer and dark layer described above.

### 3.2. Sublayer Evolution Driven by Plastic Deformation

When the grinding heat input is insufficient to trigger significant phase transformation, plastic deformation becomes the dominant mechanism driving metamorphic layer formation. The extremely high strain rates and severe plastic strain in the grinding contact zone establish steep strain and strain-rate gradients from the workpiece surface inward. These gradients drive a cascade of microstructural evolutions—dislocation evolution, dynamic recovery (DRV), and DRX—ultimately producing a gradient grain refinement layer [8,53,61,65]. The essence of this process is a deformation-dominated gradient structure that transitions from the surface inward with decreasing strain and strain rate: from a nanocrystalline zone at the outermost surface, through a submicron/fine-grained intermediate zone, to a dislocation structure zone at depth, and finally to the unaffected bulk substrate [66,67].

In the early stage of high-strain-rate plastic deformation, dislocations multiply rapidly and form high-density dislocation tangles within grains. As strain accumulates, dislocations rearrange through slip and climb, progressively forming low-energy configurations such as dislocation walls and dislocation cells [12,61]. Within the first few micrometers beneath the ground surface, dislocation density decreases from its peak value with increasing depth, providing the microstructural foundation for work hardening. Experimental observations show that after grinding 3J33 steel, dislocation density increases significantly and the flow stress is modulated by the competition between dislocation density and the Taylor factor. In molecular dynamics simulations of polycrystalline iron, lattice/grain rotation initiates at the surface as a stress-induced primary deformation mechanism and propagates inward via dislocation slip mediation [68,69]. Dislocation cell size increases with depth, and this gradient dislocation structure is directly linked to the subsequent grain refinement process.

Dynamic recovery constitutes the initial stage of plastic-deformation-driven microstructural evolution. Under high-strain-rate conditions, dislocations rearrange through cross-slip and climb to form subgrain boundaries, partitioning the original grains into subgrains with small misorientations. The formation of subgrain boundaries reduces the stored energy of the system, allowing the material to reach a dynamic equilibrium during deformation [70,71]. In creep-feed grinding, a complete evolutionary sequence of dislocation lines, tangles, walls, cells, and subgrain boundaries was observed at only 120 °C, with negligible thermal influence, confirming that this sequence is driven purely by plastic deformation [66]. Similar dislocation structure gradients were observed in cryogenic grinding of 300M ultra-high-strength steel, where martensitic and austenitic transformations were effectively suppressed by the low temperature, leaving dislocation activity as the sole driving force for microstructural refinement [67].

With further increases in strain and temperature, dynamic recrystallization becomes the dominant grain refinement mechanism. Continuous dynamic recrystallization (CDRX) proceeds through progressive subgrain rotation and the gradual transformation of subgrain boundaries into high-angle grain boundaries (HAGBs) as they absorb dislocations, ultimately forming new equiaxed grains [70,72]. FIB+TKD characterization of the grinding subsurface has directly confirmed this CDRX subgrain rotation mechanism: high-density dislocations nucleate at stress concentrations at two-phase interfaces, forming subgrain boundaries (SGBs) with continuously accumulating misorientation; low-angle grain boundaries (LAGBs) transition to HAGBs when the misorientation reaches approximately 15°, marking the formation of new grains with significantly reduced dislocation density [72]. Grinding strengthening experiments on 40Cr alloy steel, employing split Hopkinson pressure bar (SHPB) constitutive modeling and the Read–Shockley equation, independently confirmed the 15° HAGB criterion [70]. Pre-stressed dry grinding studies indicate that the pre-stress state alters the driving force for dislocation motion, thereby influencing subgrain formation and evolution. Appropriate pre-stress promotes dislocation multiplication, slip/climb/recombination, subgrain formation, misorientation increase to HAGB levels, and DRX nucleation, resulting in grain refinement [73]. It should be noted that, in contrast to CDRX, discontinuous dynamic recrystallization (dDRX)—which proceeds through local grain-boundary bulging and nucleation—makes a smaller contribution under relatively low strain or temperature conditions.

**Figure 5:**
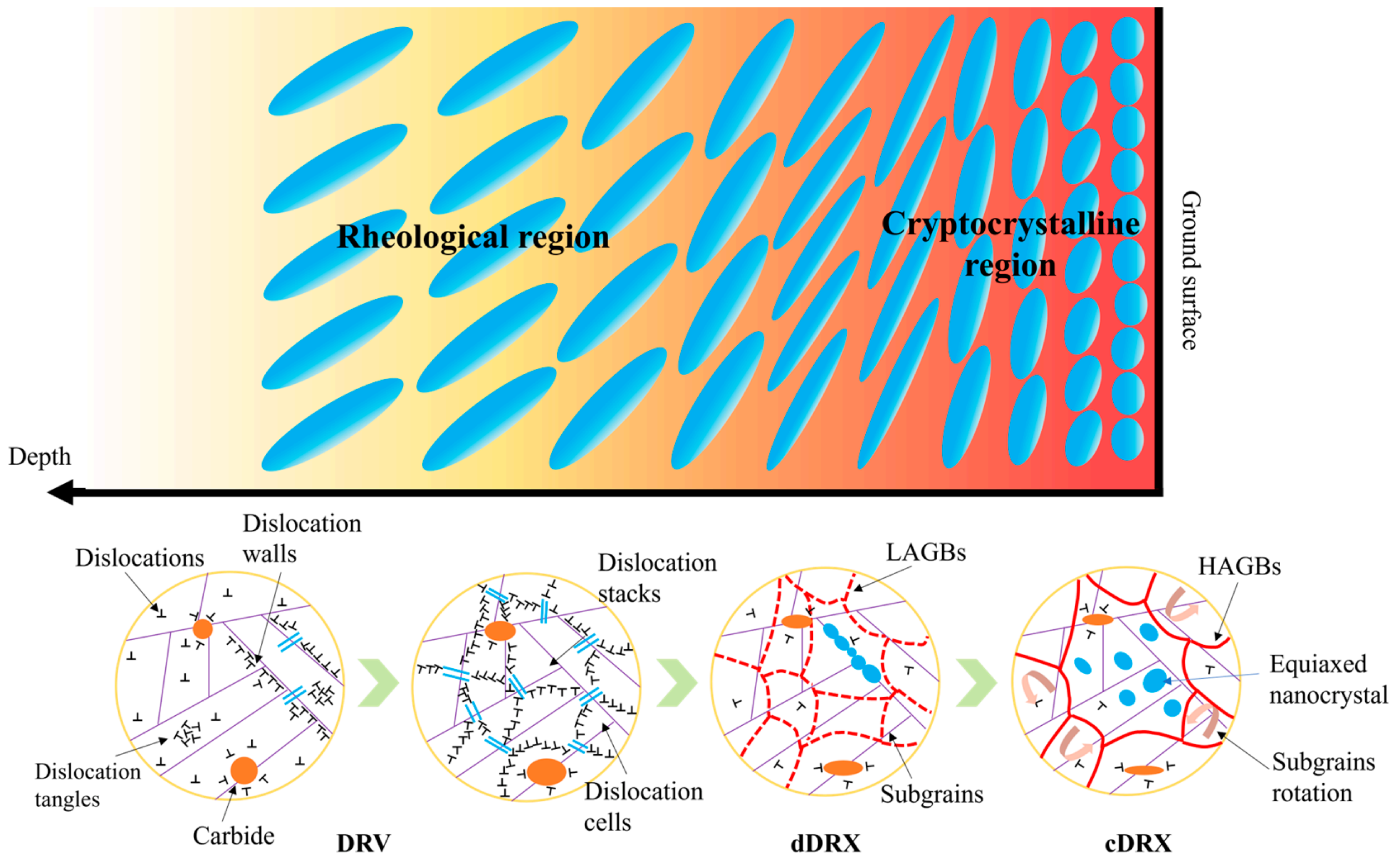
Schematic illustration of nanocrystallization at different stages, with depth decreasing from left to right.

During nanocrystallization of the grinding metamorphic layer, CDRX and dDRX exhibit a competitive synergistic relationship [61]. In the surface layer, where strain and temperature are highest, CDRX dominates, producing a uniform equiaxed nanocrystalline structure. In the transition zone, both mechanisms coexist, with CDRX prevailing in the surface region and dDRX contributing increasingly at greater depth [71]. This competitive grain refinement pathway has been validated across multiple materials and constitutes the fundamental model of grinding-induced nanocrystallization [65]. Figure 5 provides a detailed schematic of the nanocrystallization evolution stages as a function of depth.

The work hardening effect resulting from grain refinement is a major contributor to the mechanical property changes in the grinding metamorphic layer. Surface mechanical grinding treatment (SMGT) studies reveal that when the equivalent strain exceeds 10–24, the boundary spacing saturates at 100–200 nm and the HAGB fraction reaches a plateau, reflecting dynamic equilibrium between defect generation and recovery [74]. SMGT has successfully produced continuous gradient structures from nanocrystalline to coarse grains in various material systems. Inconel 625 developed a 600 μm thick gradient nanotwinned layer after SMGT [75]. Maraging steel subjected to SMGT followed by heating yielded an ultra-hard surface layer (8.9 GPa) with a continuous gradient transition to the substrate—lacking a distinct interface—unlike the abrupt interfaces of conventional coatings and surface modification layers [76]. Molecular dynamics simulations of polycrystalline iron show that high subsurface stresses induce large-angle lattice/grain rotation, forming new grain boundaries and grains, while deeper regions are dominated by dislocation nucleation, multiplication, and slip, with minor deformation twinning under transient high stress [68]. EBSD and TEM analyses of surface-modified 300M steel confirm a direct correspondence between depth-dependent dislocation structures and the decreasing hardness profile [77]. The synergistic action of these multi-scale deformation mechanisms enables the grinding metamorphic layer to achieve high strength while maintaining good toughness.

### 3.3. Sublayer Evolution Under Thermal–Mechanical Coupling

**Figure 6:**
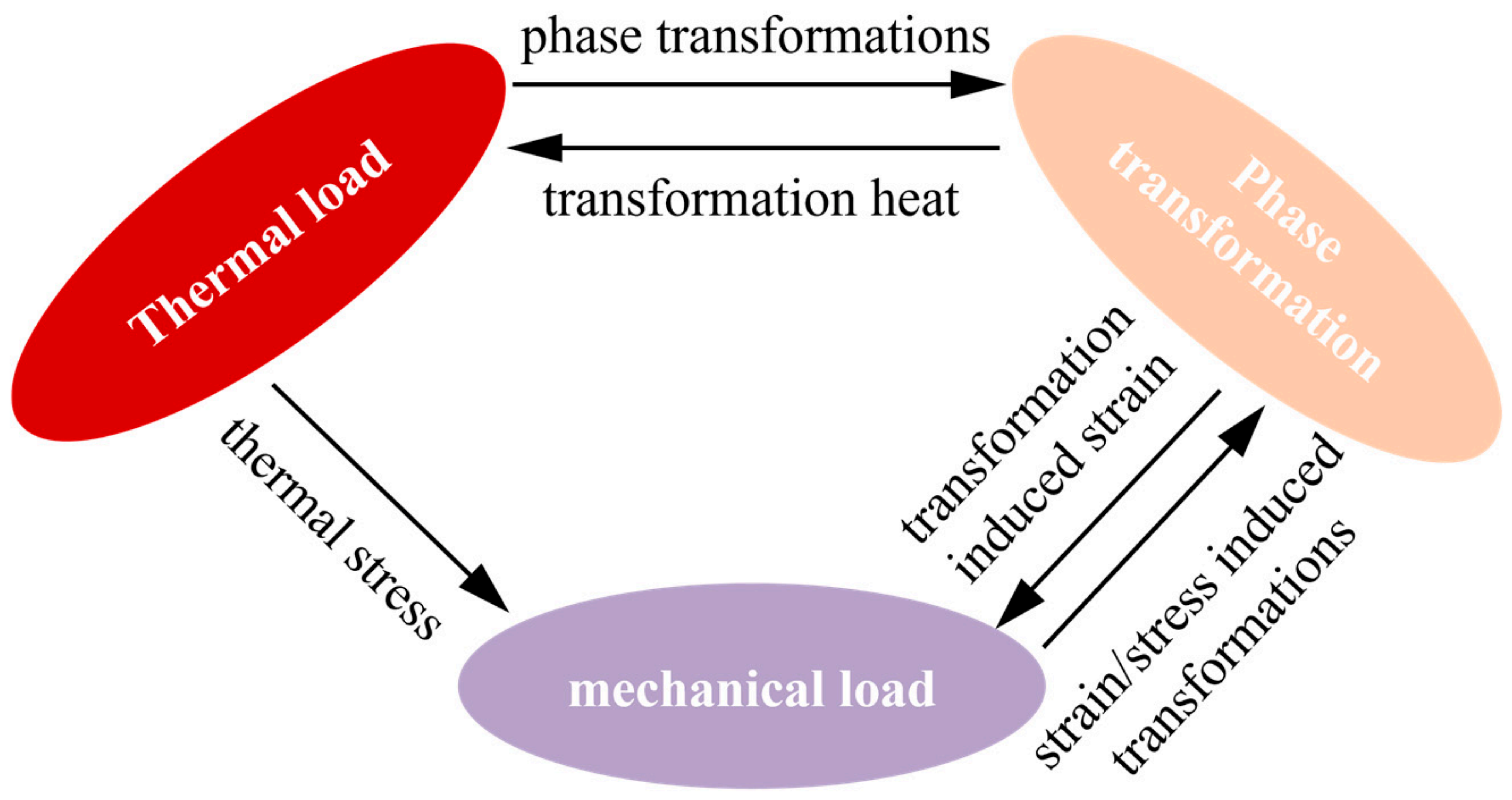
Schematic of the multiphysics process with inter-field coupling interactions during grinding [82].

Section 3.1 and Section 3.2 examined sublayer evolution driven independently by phase transformation and plastic deformation, respectively. Under real grinding conditions, however, these two mechanisms do not operate independently. The formation of the metamorphic layer is inherently a nonlinear process involving the coupled interaction among the temperature field, stress field, phase transformation, and microstructure [78,79]. On one hand, grinding heat reduces the yield strength of the surface layer, inducing thermal softening that concentrates plastic deformation toward the surface. On the other hand, the mechanical work of plastic deformation is converted into deformation heat, which feeds back into the thermal field, elevating and reshaping the temperature distribution [16,20]. Mahdi and Zhang [80] established an early finite element framework coupling thermal, plastic, and phase-transformation effects in grinding, demonstrating that the subsurface temperature field is influenced not only by heat conduction but also significantly by deformation heat from plastic work. They further showed that when materials undergo phase-transformation hardening or martensitic volume expansion, the magnitude of residual stress—whether tensile or compressive—is amplified overall. Consequently, the residual stress distribution under coupled conditions systematically deviates from predictions based on purely thermal or purely mechanical loading [81]; neither thermal nor mechanical criteria alone are sufficient to describe metamorphic layer formation. Thus, the process constitutes a multiphysics problem in which physical fields interact through coupling, as illustrated in Figure 6.

The thermal–mechanical coupling in grinding can be quantitatively described through a three-field coupling framework. Considering first the temperature field, it is typically based on Jaeger’s moving heat source theory, in which the grinding zone heat input is modeled as a rectangular or triangular heat source moving along the workpiece surface at the feed rate [8]:

(17) $$\theta \left(X,Y,Z\right)=q_{p}/\left(2\pi kR\right)\cdot exp\left[-v\left(R+X\right)/\left(2a\right)\right],$$

(18) $$R=\sqrt{\left(X^{2}+Y^{2}+Z^{2}\right)},$$

where $\theta \left(X,Y,Z\right)$ is the temperature rise at spatial coordinates $\left(X,Y,Z\right)$, $q_{p}$ is the total power of the moving point heat source, k is the thermal conductivity of the workpiece material, R is the Euclidean distance from the heat source origin to the calculation point, X,Y,Z are coordinates in the moving coordinate system attached to the heat source, v is the moving velocity of the heat source equal to the workpiece feed rate, and a is the thermal diffusivity of the workpiece material.

Under a two-dimensional simplification, the temperature rise at any point within the workpiece can be obtained by integrating the Green’s function of the moving heat source along the contact arc length. The heat flux amplitude is determined by the grinding power, the contact arc length, and the heat partition ratio into the workpiece. It is worth emphasizing that the mechanical work consumed by the grinding force constitutes the primary heat source; therefore, the tangential grinding force directly enters the heat flux expression. Additionally, the latent heat of phase transformation is superimposed onto the temperature field as an internal heat source, coupling the temperature field with the phase transformation process.

The microstructure field comes next. To quantitatively describe diffusion-controlled transformations, the Johnson–Mehl–Avrami (JMA) model is commonly employed [83]. Assuming a zero nucleation rate—all nuclei form at t = 0 and the transformation rate is controlled solely by growth—the model simplifies to

(19) $$p=1-exp\left(-\eta^{n}\right),$$

(20) $$\eta =\frac{K}{\beta }\int_{T_{0}}^{T}\;exp\left(-\frac{Q}{RT}\right)dT,$$

(21) $$K=gN_{0}k_{0},$$

where p is the fraction of the new phase, η is the non-isothermal transformation integral variable, n is the Avrami exponent, K is the lumped kinetic constant, β is the heating rate, $T_{0}$ is the initial temperature for phase-transformation integration, T is the instantaneous temperature, Q is the activation energy for phase transformation, R is the universal gas constant, g is the geometric growth factor, $N_{0}$ is the number of pre-existing nuclei, and $k_{0}$ is the exponential factor. The advantage of this approach is that the model parameters Q, K, and n can be determined directly from dilatometric curves without regression analysis.

During grinding, the cooling rate—especially when coolant is applied—is typically high enough to drive austenite-to-martensite transformation, provided the surface region has been austenitized. The Koistinen–Marburger (K–M) model describes the martensite fraction $p_{m}$ as a function of temperature T:

(22) $$p_{m}=p_{max}\left(1-exp\left(-\frac{M_{s}-T}{b}\right)\right),$$

where $p_{max}$ is the maximum possible martensite fraction, the constant b is determined as 70 °C through dilatometry, $M_{s}$ is the martensite start temperature, and T is the instantaneous temperature. During short-duration austenitization, cementite may only partially dissolve, so the austenite carbon content depends on the peak temperature reached and the austenitization time.

The JMA equation relates the transformed fraction of diffusion-controlled transformations to the temperature–time history, with the Avrami exponent and activation energy determinable from dilatometric experiments at varying rates. The K–M equation expresses the diffusionless martensite fraction as an exponential function of undercooling [82]. The critical coupling point is that the $M_{s}$ temperature in the K–M equation is not constant but is significantly influenced by the stress state and the chemical composition of the austenite [56,82], which is precisely where the mechanical field enters the phase transformation framework.

It should be noted that more advanced phase transformation models exist, such as the cellular automaton (CA) approach [58,84] and the Leblond phenomenological model [77]. The CA model offers higher spatial resolution for microstructure prediction and will be further discussed in Section 5.5 in the context of cooling rate effects on martensite grain size. However, the JMA model remains the standard choice in the grinding FE literature because their parameters can be directly calibrated from standard dilatometric experiments and they offer the computational efficiency required for integration within coupled thermal–mechanical–phase-transformation simulations at the component scale.

The coupled thermal–mechanical interaction generates a total strain tensor $\varepsilon_{ij}^{t}$ that varies with both time and position:

(23) $$\varepsilon_{ij}^{t}=\varepsilon_{ij}^{el}+\varepsilon_{ij}^{pl}+\varepsilon_{ij}^{th}+\varepsilon_{ij}^{tr}+\varepsilon_{ij}^{tp},$$

The elastic strain $\;\varepsilon_{ij}^{el}$, plastic strain $\varepsilon_{ij}^{pl}$, thermal strain $\varepsilon_{ij}^{th}$, phase transformation strain $\varepsilon_{ij}^{tr}$, and transformation-induced plasticity (TRIP) strains $\varepsilon_{ij}^{tp}$ are assumed to be independent and of small magnitude. The latter two components are the hallmarks of the coupling. The phase transformation volume strain originates from the specific volume difference between parent and product phases, generating a compressive stress contribution in the surface layer [80]. TRIP describes the additional plastic flow that occurs during ongoing phase transformation under an applied stress well below the yield strength of either phase; its magnitude is proportional to the transformation rate and the deviatoric stress tensor. Rajaei et al. demonstrated in a fully coupled thermal–mechanical–metallurgical FE model of 52100 grinding that, without accounting for these two terms, the experimentally measured residual stress distribution could not be reproduced—confirming them as essential components of the coupling.

Plastic strain is determined using the von Mises criterion. The flow stress of AISI 52100 is defined through a power-law hardening model (Equation (24)), and the flow stress of the homogenized multiphase microstructure is approximated via a rule-of-mixtures formula (Equation (25)) [79,85]:

(24) $$\sigma_{f}^{\left(\varphi \right)}=\sigma_{y}^{\left(\varphi \right)}+k\cdot \varepsilon_{pl}^{n},$$

(25) $$\sigma_{F}=f(P_{\alpha })\sigma_{f}^{\left(\alpha \right)}+(1-f(P_{\alpha }))\sigma_{f}^{\left(\gamma \right)},$$

$\sigma_{f}^{\left(\varphi \right)}$ and $\sigma_{y}^{\left(\varphi \right)}$ are the flow stresses of phase φ with and without work hardening, respectively. $\sigma_{f}^{\left(\alpha \right)}$ and $\sigma_{f}^{\left(\gamma \right)}$ are the flow stresses of phases α and γ, respectively. k and n are the hardening parameters; $\sigma_{F}$ is the flow stress of the multiphase mixed microstructure; and $f(P_{\alpha })$ is the weighting factor from reference [85], where $P_{\alpha }$ is the sum of the volume fractions of all body-centered cubic (ferritic) phases. Thermal strain, phase transformation strain, and TRIP strain are all modeled based on dilatometric experiments. The density of the dilatometric specimen is measured prior to testing as a reference baseline $\rho (T_{0})$. The temperature-dependent density during heating, cooling, and phase transformation can then be determined by correlating the single-phase thermal expansion coefficients, where each single-phase thermal expansion coefficient is defined as the slope of the corresponding thermal expansion curve, as given by Equation (26).

(26) $$\rho (T)=\rho (T_{0})exp\left(-\int_{T_{0}}^{T}\;3\cdot \alpha (T)dT\right),$$

where ρ(T) is the temperature-dependent density, $\rho (T_{0})$ is the reference density at reference temperature $T_{0}$, and α(T) is the temperature-dependent linear thermal-expansion coefficient.

Equation (27) links thermal strain, phase transformation strain, and density change [86]:

(27) $$\varepsilon^{th}+\varepsilon^{tr}=\sqrt[{3}]{\frac{\rho (T_{0})}{\rho (T)}}-1,$$

During solid-state phase transformation, even when the applied equivalent stress is below the yield stress of both parent and product phases, additional plastic flow—transformation-induced plasticity—occurs under internal or external stresses. This cannot be described by the von Mises criterion. The present review adopts the Fisher model (Equation (28)) [85,87] to characterize the TRIP effect:

(28) $$\varepsilon_{ij}^{tp}=\frac{3}{2}KS_{ij}\cdot (1-ln(p))\cdot p,$$

where K is a constant, $S_{ij}$ is the deviatoric stress, and p is the transformed fraction.

The three fields close into a cycle through the constitutive equations: the temperature field provides the driving force for phase transformation; phase transformation alters the local stress state and material properties; and the stress state, in turn, influences the $M_{s}$ temperature and phase transformation kinetics. The thermal–mechanical coupling pathways during grinding include deformation heat feedback, stress-mediated regulation of phase transformation thermodynamics, and the synergistic thermal–mechanical evolution of carbides.

Under high-strain-rate grinding, the heat generated by plastic deformation cannot dissipate into the bulk within millisecond timescales, causing a local temperature surge. This temperature rise reduces the flow stress, further concentrating deformation in the already softened region and generating more plastic strain and deformation heat—a positive feedback loop [81]. Liu et al. [88] introduced a thermal–mechanical coupling coefficient $\zeta =Tmax/F_{t}$ in abrasive belt grinding of titanium alloys to quantify the transition from mechanically dominated to thermally dominated regimes. Although their material system differs from bearing steel, the coupling coefficient concept is transferable to describing the relative weights of grinding force and heat. This thermal feedback raises the actual peak surface temperature above predictions considering only external heat sources, directly amplifying thermal effects and deepening sublayer influence.

**Figure 7:**
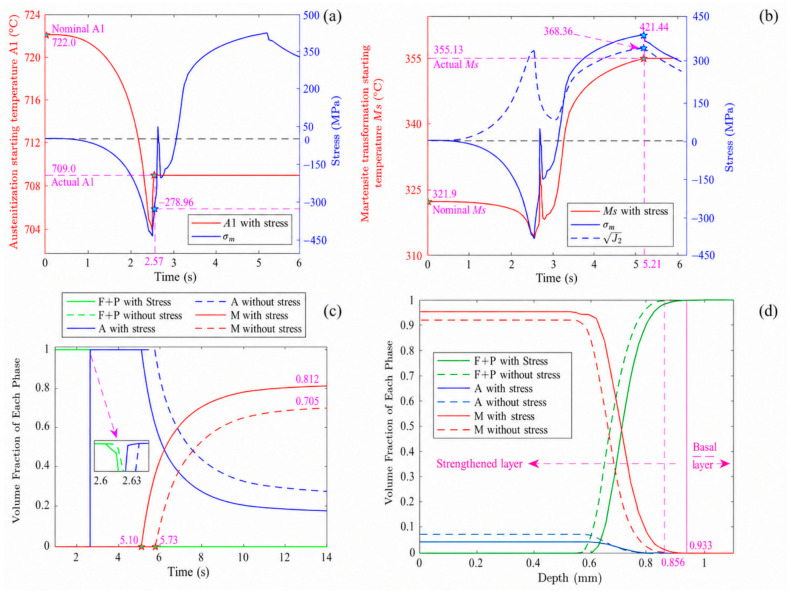
Simulation results showing the effect of stress on grinding phase transformation temperatures, stress history, phase evolution, and hardened layer microstructure distribution [89]. (**a**) Hydrostatic pressure history and stress-modified $A_{1}$ temperature; (**b**) hydrostatic and equivalent stress histories, and stress-modified $M_{s}$ temperature; (**c**) effect of stress on the evolution of each phase and the stars represent the start time of phase transformation; (**d**) effect of stress on the microstructure distribution and thickness of the grinding metamorphic layer.

Grinding stress alters the phase transformation critical temperatures by modifying the free energy difference between parent and product phases—the most essential coupling pathway through which the mechanical field intervenes in phase transformation. Xiu conducted a fully coupled finite element analysis of grind-hardening in 1045 steel [89]. Prior to austenitization, the predominantly hydrostatic compressive stress reduces $A_{c1}$ from 722 °C to 709 °C (≈13 °C drop), enabling austenitization at lower temperatures (Figure 7a). Prior to martensitic transformation, the stress state—dominated by hydrostatic tension combined with the second invariant of deviatoric stress—raises $M_{s}$ from 321.9 °C to 355.1 °C, causing martensite to form earlier and more extensively during cooling (Figure 7b). Figure 7c compares phase evolution with and without stress effects: in both cases, complete austenitization occurs (100% austenite), but the onset of austenite transformation is earlier when stress is included because hydrostatic compressive stress lowers the $A_{1}$ transformation temperature. Figure 7d shows that the hardened layer thickness reaches 933 μm with stress effects versus 856 μm without, indicating that accounting for stress enables phase transformation at lower temperatures and greater depths.

This stress effect is distributed non-uniformly along the grinding contact arc: compressive stress ahead of the wheel favors $M_{s}$ elevation and early martensitic transformation, while the tensile zone behind the wheel has the opposite effect [16,90]. In high-speed hard turning of 52100, Duan employed the Clausius–Clapeyron relation to convert equivalent stress and strain energy density into the actual transformation temperature, revealing that white layer thickness first increases then decreases with cutting speed, followed by a monotonic increase with flank wear [91]. Han further showed in 1045 steel that even when the net temperature rise is well below the nominal $A_{c1}$, contact stresses of 355–662 MPa, which is estimated via the Clausius–Clapeyron equation, can lower the transformation temperature by approximately 45 °C, and combined with plastic-deformation-enhanced carbon diffusion, are sufficient to induce white layer formation [78,92]. A critical distinction should be made: the upward shift in transformation temperature due to heating rate [79,82] and the downward shift due to stress [89,91,92] are mechanistically different effects and should be accounted for separately. This stress-mediated regulation ultimately manifests in the residual stress state. In hard cutting of 300M steel, mechanical plowing contributes compressive stress while grinding heat contributes tensile stress; their competition enables surface residual stress to vary from −703 to +202 MPa, consistent with the integrated modeling results of Wang et al. for bearing ring raceway grinding [16,93].

The evolution of carbides under thermal–mechanical coupling is generally attributed to thermal dissolution and mechanical fragmentation, but the actual behavior strongly depends on carbide size. As discussed in Section 3.1, nanoscale carbides dissolve rapidly within millisecond thermal cycles due to their short diffusion distances, providing the primary carbon source for surface carbon supersaturation and retained austenite recovery. In contrast, submicron- to micron-scale secondary (Fe,Cr)_3_C carbides—whose dissolution is limited by the slow diffusion of substitutional Cr atoms—cannot dissolve completely within the extremely short contact time of grinding or hard machining. Experimental characterization and DICTRA simulations by Hosseini et al. [94] of such carbides in the white layer of hard-turned 52100 steel consistently show that, even at 1000 °C with a dwell time of 100–500 μs, carbide diameter decreases by less than 2 nm; measurable dissolution requires approximately 1300 °C. Thus, complete dissolution and reprecipitation at this scale is not feasible within this time window. Elevated hydrostatic pressure stabilizes the austenite + cementite two-phase region and inhibits dissolution, even though the equivalent contact pressure, via the Clausius–Clapeyron relation, can lower $A_{c1}$ by approximately 100 °C to promote austenitization. In contrast to their limited dissolution, these larger carbides respond primarily through plastic deformation.

## 4. Characterization of the Grinding Metamorphic Layer in Bearing Steel

### 4.1. Microstructural Characteristics of Ground Surface Sublayers

For martensitic hardened bearing steels, the grinding metamorphic layer exhibits a gradient sublayer architecture along the depth direction, comprising a white layer, a dark layer, and a transition zone [95]. The white layer occupies the outermost surface and consists of an etching-resistant, highly refined cryptocrystalline/nanocrystalline martensitic structure containing elevated levels of high-carbon retained austenite, refined or partially dissolved carbides, and a high density of dislocations. In contrast to conventional tempered martensite, it features a misoriented cellular structure on the order of 100 nm. Microhardness measurements on hardened GCr15 grinding surfaces further confirm that the white layer hardness increases to approximately 1000 HV. Beneath the white layer lies the dark layer—an over-tempered zone induced by grinding heat at temperatures below the full austenitization threshold. Its microstructure consists of tempered troostite, tempered martensite, and fine precipitated carbides, with a hardness lower than that of the base material, forming a softened band of approximately 50 μm width with hardness below 500 HV [29]. The transition zone, situated between the dark layer and the bulk substrate, exhibits continuous gradient transitions in grain size, dislocation density, and hardness with depth, eventually converging to the properties of the unaffected base material, reflecting the gradient attenuation of the grinding temperature and stress fields along the depth direction.

The clarity and thickness of the sublayer architecture depend strongly on the carbide type and thermal stability of the bearing steel grade. AISI 52100 steel is dominated by M_7_C_3_-type and cementite-type carbides, which possess relatively low thermal stability and are more readily dissolved under grinding temperatures, thereby favoring the formation of white layers with well-defined boundaries whose thickness increases markedly with grinding depth. In GCr15 steel, the maximum white layer thickness increases from approximately 4 μm at a grinding depth of 0.01 mm to approximately 35 μm at 0.08 mm [96]; when the grinding depth exceeds a critical threshold of approximately 25 μm, the white layer transitions from a discontinuous distribution to a continuous, dense band [10]. In contrast, M50 steel contains highly thermally stable MC/M_2_C-type (V, Mo) carbides, which undergo only partial dissolution under grinding temperatures and extremely short contact times, remaining appreciably within the metamorphic layer [97]. Consequently, no clear white layer–dark layer interface develops; instead, the metamorphic layer is described by a gradient classification comprising an acicular martensite zone, a severe plastic deformation zone, and a mixed zone [19]. This type of metamorphic layer is also thinner overall—for 8Cr4Mo4V steel, the metamorphic layer thickness is on the order of 30 μm [18], with the dense plastic deformation zone measuring only approximately 5 μm [19]. These comparisons underscore that differences in carbide type and thermal stability are the fundamental material-level factors driving the divergence in sublayer morphology and gradient characteristics across bearing steel grades.

It should be noted that the three-layer classification is most clearly applicable to low-alloy bearing steels (e.g., AISI 52100, GCr15) under thermally dominated grinding conditions, where M_7_C_3_ carbides dissolve readily and the phase-transformation-driven white layer and tempering-induced dark layer produce distinct etching contrast. For high-alloy steels such as M50 and 8Cr4Mo4V, where MC/M_2_C carbides exhibit high thermal stability and the affected layer forms under stronger mechanical contribution, the three-layer scheme does not apply cleanly. Alternative classifications, such as the acicular martensite zone, severe plastic deformation zone, and mixed zone proposed by Zhang et al. [96], or the large plastic deformation layer, acicular-martensite-free layer, and mixed layer by Liu et al. [10], have been developed to better capture the gradient deformation characteristics of these materials. The choice of classification therefore reflects not scholarly disagreement but rather material-dependent and mechanism-dependent differences in the affected layer’s physical nature.

### 4.2. Microstructural Morphology and Phase Structure Analysis of the Metamorphic Layer

#### 4.2.1. Characterization of the Multilayer Sublayer Structure

The primary task in characterizing the multilayer structure of the metamorphic layer is to identify and delineate the geometric boundaries of the white layer, dark layer, and transition zone, and to track their evolution with depth. Because the temperature and stress fields in the grinding contact zone vary continuously along the depth direction, the sublayers are inherently gradient transitions rather than abrupt interfaces. No single technique is capable of simultaneously providing morphological, compositional, and crystallographic information; consequently, optical microscopy (OM), scanning electron microscopy (SEM), and electron backscatter diffraction (EBSD) are typically used in combination.

**Figure 8:**
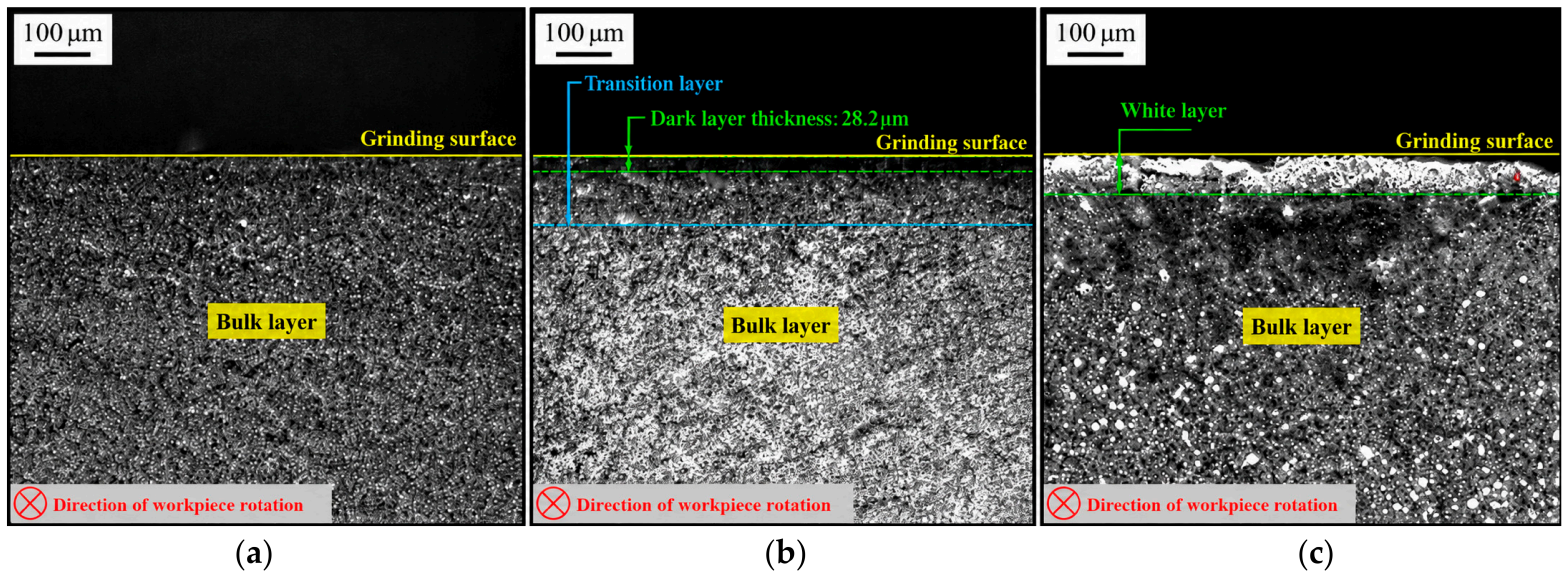
OM micrographs of the metamorphic layer [48]. (**a**) Surface metallography without an obvious metamorphic layer; (**b**) cross-sectional metallography of a specimen with a single tempered dark layer; (**c**) surface metallography showing both a white layer and a tempered dark layer.

OM clearly resolves the three-layer architecture: the etching-resistant white layer at the outermost surface, the darker-appearing subsurface dark layer, and the unaffected base material. In the annealed condition of the same steel grade, only the white layer appears after grinding without a dark layer, confirming that the dark layer is fundamentally a product of over-tempering induced by grinding heat [13]. Owing to the non-uniform instantaneous temperatures and strains arising from the random distribution of abrasive grains, the white layer often exhibits a discontinuous distribution with significant thickness fluctuations along the grinding direction. In ground GCr15 cross-sections, the maximum white layer thickness increases from approximately 4 μm to 35 μm with increasing grinding depth, while the dark layer can reach thicknesses exceeding 100 μm [96,98]. Once the grinding depth surpasses a critical threshold, the white layer transitions from a discontinuous to a continuous dense band, accompanied by the appearance of the dark layer [10]. In conical roller bearing outer ring inner raceway grinding, the tapered wall thickness variation creates non-uniform temperature fields; OM observations reveal a pronounced variation in dark layer thickness along the raceway width direction. Figure 8a shows the OM microstructure of a specimen ground at low speed and shallow depth, exhibiting no metamorphic layer, where the grinding arc temperature remained below the tempering threshold. In Figure 8b, a uniformly tempered dark layer with an average thickness of 28.2 μm has formed; grinding heat induced only martensite tempering without reaching the phase transformation temperature, and the retained austenite did not undergo transformation. In Figure 8c, a discontinuous white layer appears at the surface, originating from the instantaneous high temperatures and strain variations caused by random abrasive grain action, which produce thickness fluctuations in the white layer. The limitation of OM lies in its insufficient spatial resolution to resolve submicron structures and its lack of adequate contrast for carbide morphology, necessitating further characterization by SEM and EBSD.

**Figure 9:**
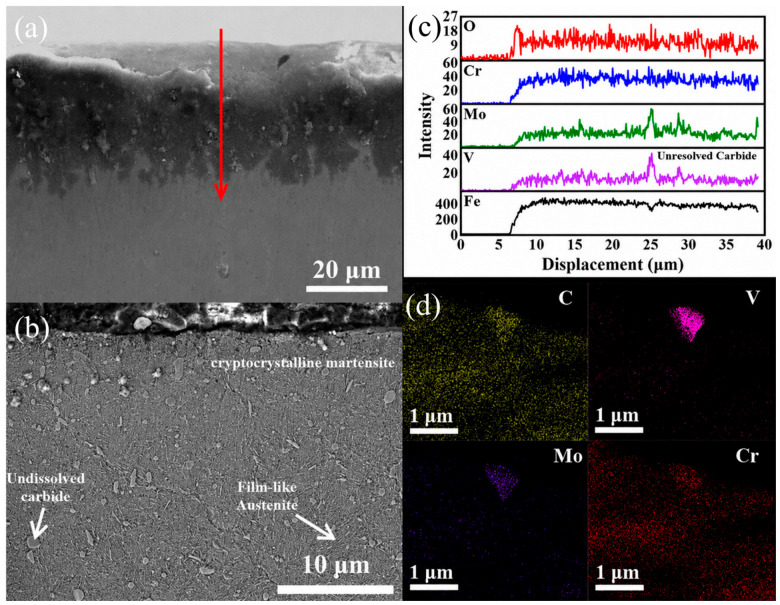
Microstructure of the grinding metamorphic layer in 8Cr4Mo4V steel at a grinding depth of 10 μm and feed rate of 40 mm/s [18]. (**a**) SEM morphology of the unetched metamorphic layer; (**b**) SEM morphology of the etched metamorphic layer, showing phase distribution; (**c**) elemental content along the red arrow line-scan; (**d**) EDS mapping of C, V, Mo, and Cr across the metamorphic layer.

The backscattered electron (BSE) mode of SEM, which leverages atomic number contrast, enables clear differentiation between carbides and the martensitic matrix, thereby revealing carbide refinement/dissolution in the white layer and carbide re-precipitation behavior in the dark layer. For low-alloy bearing steels, SEM characterization reveals that the degree of carbide dissolution is governed by the combined constraints of carbide thermal stability and grinding contact time; carbides in the white layer are not always completely dissolved. Figure 9 presents the results of elemental line-scan analysis across the grinding metamorphic layer of 8Cr4Mo4V high-alloy bearing steel [18]. Figure 9a shows a cross-sectional morphology perpendicular to the ground surface, where the subsurface grinding metamorphic zone can be distinguished from the interior base material; the red line marks the elemental line-scan path perpendicular to the machined surface, used to capture compositional variations along the depth direction. Building on this, the etched cross-sectional microstructure in Figure 9b further reveals the multiphase composite structure within the metamorphic layer. The intense plastic deformation and transient high temperatures of grinding jointly produce nanocrystalline cryptocrystalline martensite as the matrix phase, while film-like retained austenite persists at grain boundaries. Meanwhile, granular undissolved Mo–V alloy carbides, precipitated during prior heat treatment and resistant to dissolution under the transient grinding thermal field, remain stably within the microstructure. The EDS line-scan results in Figure 9c show that the oxygen content is highest at the outermost surface and gradually decreases inward; Cr is distributed uniformly across the entire scanned region; Mo and V signals exhibit coincident local peaks corresponding to carbide locations; and the Fe signal intensity in the surface oxide layer is markedly lower than in the base material. Figure 9d presents the elemental mapping of a single carbide particle, where C, Mo, and V are synchronously enriched at the particle location while Cr shows no significant segregation, confirming that this second phase is a Mo–V alloy carbide [18].

Thus, SEM-BSE/EDS is capable of characterizing the complete gradient sequence of carbide behavior during grinding across the metamorphic layer. Furthermore, the compositional gradient aligns with the white layer/dark layer interface position identified by OM, providing complementary compositional evidence for the analysis of the metamorphic layer.

**Figure 10:**
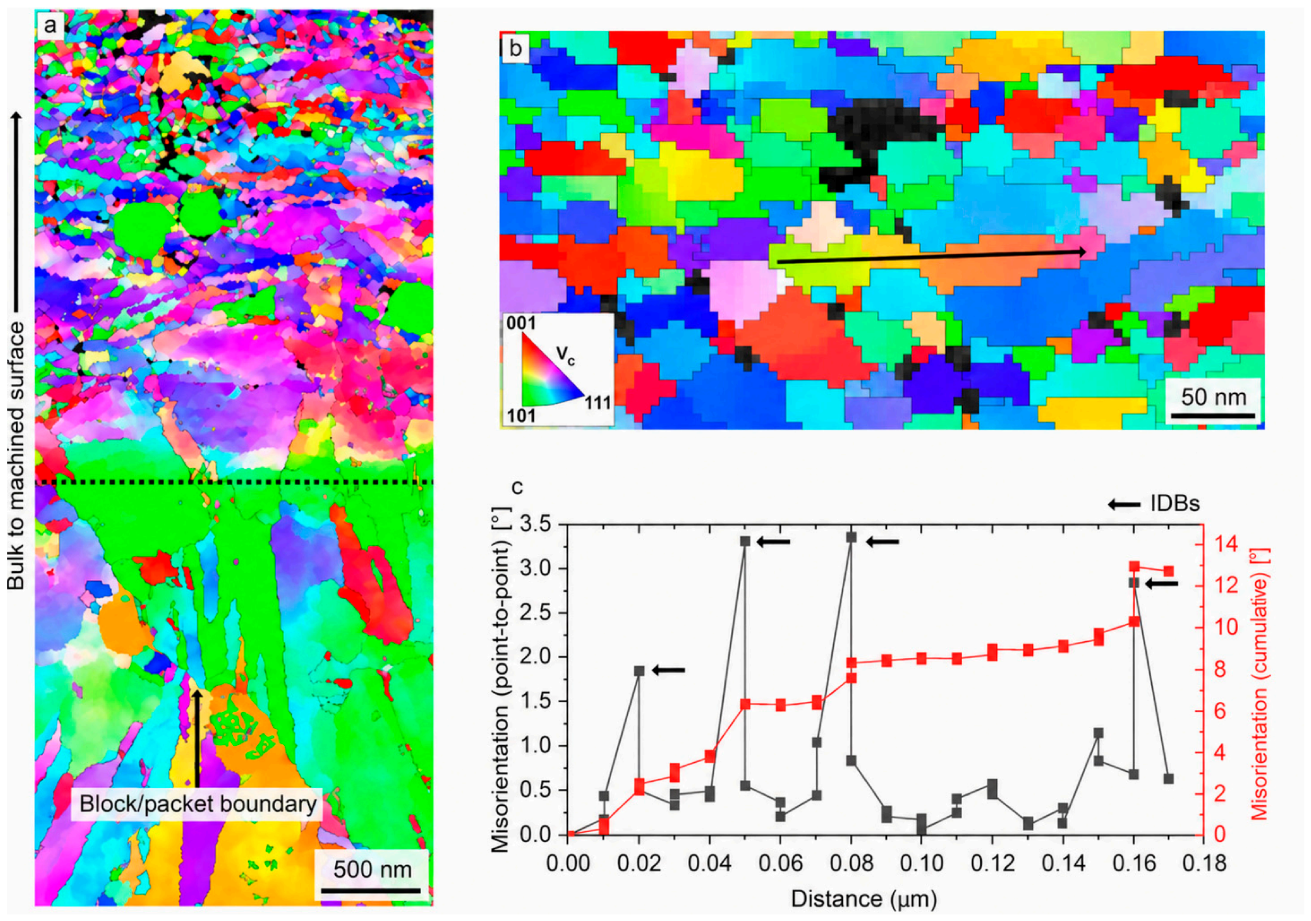
TKD characterization results of the hard-turned surface layer [65]. (**a**) Full-scale IPF orientation map from the base material to the machined surface; (**b**) high-resolution inverse pole figure of lamellar grains in the drag zone; (**c**) point-to-point and cumulative misorientation line profiles within grains, revealing the GNB and IDB dominated grain subdivision mechanism.

EBSD introduces crystallographic information into metamorphic layer characterization, tracking the gradient evolution of grain size and grain boundary misorientation along the depth direction via Kikuchi patterns, thereby linking the gradient structure of the metamorphic layer to its crystallographic evolution. Taking grind-hardened 40Cr steel as an example, EBSD combined with cellular automaton simulations consistently demonstrates that the degree of DRX decreases from the surface inward. In the surface layer, where strain and temperature are highest, DRX is fully developed, forming fine equiaxed grains dominated by high-angle grain boundaries (HAGBs). In the transition zone, DRX is incomplete and dynamic recovery dominates, with an increased proportion of subgrain boundaries. At greater depths, only thermal effects are present without significant plastic deformation. The DRX volume fraction decreases from approximately 95% at the surface to 30–60% at a depth of approximately 90 μm. Proeutectoid grain size increases from approximately 5 μm at the surface to approximately 21 μm at a depth of approximately 100 μm, and the refined layer penetration depth ranges from approximately 76 to 106 μm [71]. Increasing grinding depth raises the HAGB fraction and promotes more complete recrystallization [70,99]. Figure 10 shows the grain subdivision and nanocrystalline white layer formation mechanism induced by hard turning of AISI 52100 steel, as revealed by transmission Kikuchi diffraction (TKD). In Figure 10a, the TKD inverse pole figure (IPF) presents the microstructural gradient from the base material to the machined surface: the base material retains a complete martensite block/lath structure, while within the material drag zone near the surface, grains undergo severe subdivision with highly randomized orientations. Figure 10b shows a high-resolution IPF of a typical lamellar grain within the drag zone, containing numerous low-angle incidental dislocation boundaries (IDBs). Misorientation line-scanning along the black horizontal line in Figure 10b is presented in Figure 10c. The black point-to-point misorientation curve exhibits multiple peaks corresponding to the low-angle boundaries formed by IDBs within the lamellae. The red cumulative misorientation curve rises continuously along the scan path, reaching a cumulative misorientation of up to 13°, while the grain periphery is separated by high-angle geometrically necessary boundaries (GNBs) and collectively confirms the grain subdivision mechanism under severe plastic deformation. Similar nanocrystalline surface-to-coarse-grained bulk gradient structures have been confirmed by SEM/TEM in ground surfaces of other materials; for instance, in ground low-carbon steel, the finest proeutectoid ferrite reaches approximately 17 nm, with progressive refinement through cellular dislocation structures, elongated dislocation structures, ultrafine lamellae, and nanoscale lamellae [100].

#### 4.2.2. Sublayer Phase Structure Analysis

**Figure 11:**
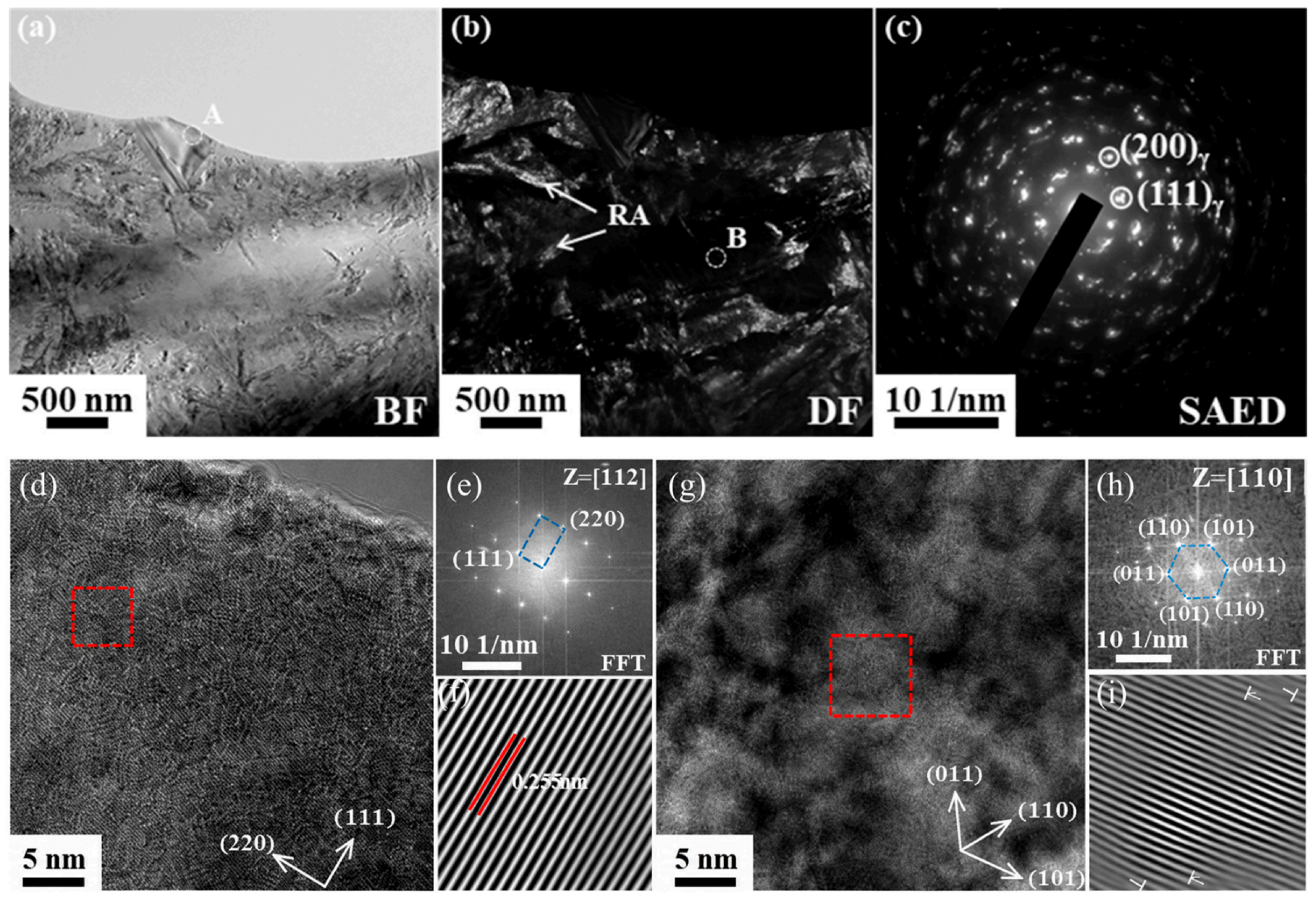
TEM characterization results of the grinding metamorphic layer in 8Cr4Mo4V steel at a grinding depth of 10 μm and feed rate of 40 mm/s [18]. (**a**) Bright-field TEM morphology of the affected layer; (**b**) dark-field TEM morphology of the metamorphic layer; (**c**) SAED pattern of retained austenite at location B in (**b**); (**d**,**g**) HRTEM images of undissolved carbides and martensite; (**e**,**h**) FFT patterns of the red boxed regions in (**d**,**g**), showing diffraction spots of undissolved carbides and martensite; (**f**,**i**) IFFT reconstructed images.

While OM, SEM, and EBSD enable macro-scale sublayer delineation and crystallographic gradient analysis, transmission electron microscopy (TEM) further determines the phase composition, grain size, crystallographic orientation, and defect structure of each sublayer at the nanoscale. TEM observations of the white layer in hardened steels reveal that, in bright-field imaging, the white layer consists of misoriented cellular structures generally smaller than 100 nm, distinctly different from conventional tempered martensite. Selected-area electron diffraction (SAED) patterns exhibit continuous or semi-continuous Debye rings, indicating extremely fine grains with nearly random orientation; the rings contain reflections from both body-centered cubic (BCC) and face-centered cubic (FCC) structures, evidencing reverse martensitic transformation during processing. The transformation of cementite reflections from discrete spots to diffuse continuous rings indicates significant carbide refinement. For bearing steels specifically, TEM characterization of the M50 grinding metamorphic layer directly reveals a phase constitution of cryptocrystalline martensite + retained austenite + undissolved alloy carbides, with a high intragranular dislocation density [18], confirming the aforementioned partial dissolution of highly stable MC/M_2_C carbides. Figure 11a shows the microstructure within the top 2 μm of the metamorphic layer in 8Cr4Mo4V steel. After heat treatment, both twin martensite and lath martensite exist in 8Cr4Mo4V steel; however, under the coupled thermal–mechanical effect of grinding, cryptocrystalline martensite forms within the metamorphic layer, accompanied by a marked change in martensite morphology. Figure 11b presents a dark-field image of the surface layer microstructure, showing retained austenite distributed in blocky and film-like morphologies within the metamorphic layer, which is consistent with the austenite morphology prior to grinding in 8Cr4Mo4V steel [6]. Figure 11c shows the SAED pattern of retained austenite, where the diffraction spot characteristics indicate refinement of the retained austenite grains. Severe plastic deformation at the ground surface substantially increases dislocation density; when the accumulated dislocation density reaches the condition for dynamic recrystallization, austenite undergoes DRX, refining grains to the nanoscale. High-resolution imaging further reveals clear, straight nanocrystalline grain boundaries with extremely high intragranular dislocation densities. The carbide types in 8Cr4Mo4V steel are primarily M_2_C and MC. HRTEM characterization and diffraction patterns identify the undissolved carbides as M_2_C type. Inverse fast Fourier transform (IFFT) reveals no dislocations within the undissolved carbides after grinding, whereas HRTEM of martensite in the metamorphic layer reveals abundant dislocations.

To achieve site-specific sampling of the white and dark layers, focused ion beam (FIB) is commonly employed to precisely select regions on cross-sections and extract TEM lamellae, thereby correlating nanoscale phase analysis with specific sublayer positions. The limitation of TEM lies in its small sampling volume and localized, qualitative observation, requiring supplementation by XRD with greater statistical representation.

**Figure 12:**
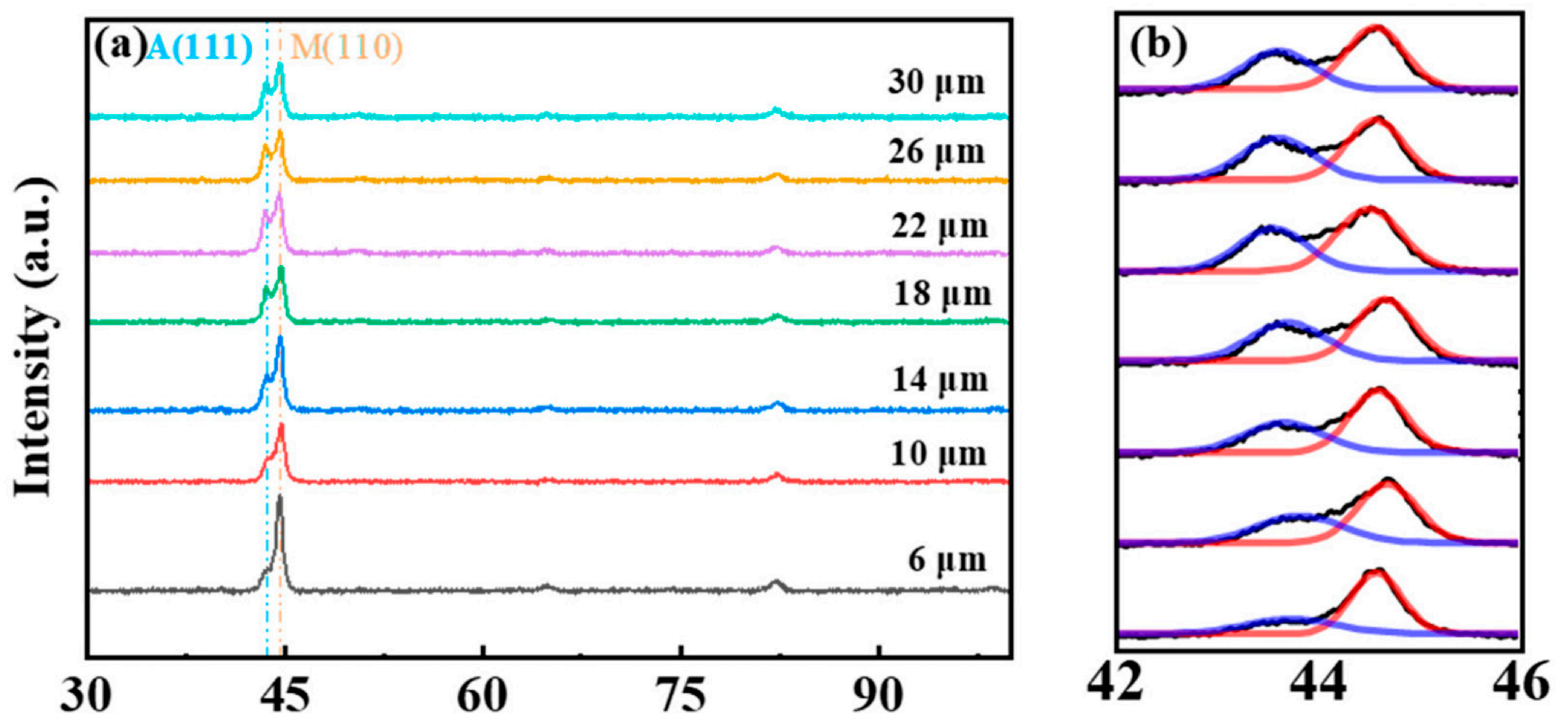
XRD analysis results of the metamorphic layer in 8Cr4Mo4V steel at a feed rate of 40 mm/s [18]. (**a**) XRD patterns at different grinding depths; (**b**) peak fitting results of the XRD patterns. The blue lines represent the peak of A(111) and the red lines denote the peak of M(110) and the black lines represent the original diffraction peak.

In contrast to the localized observations of TEM, XRD provides statistically averaged information at the sublayer scale. For phase quantification, XRD with multi-peak fitting determines the retained austenite content in the white layer, with the measured value depending on the relative contributions of thermal and mechanical effects. In the ground white layer of hardened GCr15 steel, the retained austenite content is actually lower than that of the base material, indicating that mechanically driven (strain-induced) martensitic transformation dominates [96]. By contrast, a purely thermal process raises the retained austenite to approximately 9.6%, highlighting the role of thermal effects. Thus, the retained austenite content in the white layer can itself serve as an indicator of the thermal-to-mechanical contribution ratio [13]. For high-alloy M50/8Cr4Mo4V steels, XRD/grazing-incidence XRD (GIXRD) resolves the depth-dependent austenite content and its recovery with increasing grinding heat [18,19]. The XRD depth profiling results shown in Figure 12 quantify, at a statistical scale, the evolution of the austenite-to-martensite phase ratio in the ground surface layer as a function of grinding depth. As the grinding depth increases, the intensity of the retained austenite diffraction peaks in Figure 12a gradually increases, indicating a rise in retained austenite content within the metamorphic layer after grinding. To quantitatively determine the martensite and retained austenite fractions, peak fitting was performed on the martensite (110) and austenite (111) diffraction peaks to obtain their respective integrated intensities. The fitting results are shown in Figure 12b, with all diffraction peaks achieving a fitting accuracy above 95%.

Quantification of retained austenite is important because its content and stability have contradictory implications for bearing service performance. An appropriate amount of stable retained austenite can blunt crack tips under contaminated/indentation lubrication conditions and generate compressive stress through strain-induced transformation, thereby improving rolling contact fatigue life. However, its decomposition during service or tempering, accompanied by volume expansion, can compromise the dimensional stability of bearings [41]. Beyond phase quantification, XRD/GIXRD peak width analysis provides sublayer crystallite size and micro-strain; for example, in the ground metamorphic layer of 8Cr4Mo4V, the variation in crystallite size and micro-strain with grinding parameters can be obtained [19]. The Williamson–Hall method further converts micro-strain into dislocation density, enabling quantitative evaluation of the degree of grinding-induced deformation [101]. In summary, TEM provides localized, qualitative phase and defect images, while XRD provides statistically averaged information at the sublayer scale; their combination is essential for a complete description of the phase composition and crystal structure of the metamorphic layer.

### 4.3. Mechanical Property Characterization of the Metamorphic Layer

**Figure 13:**
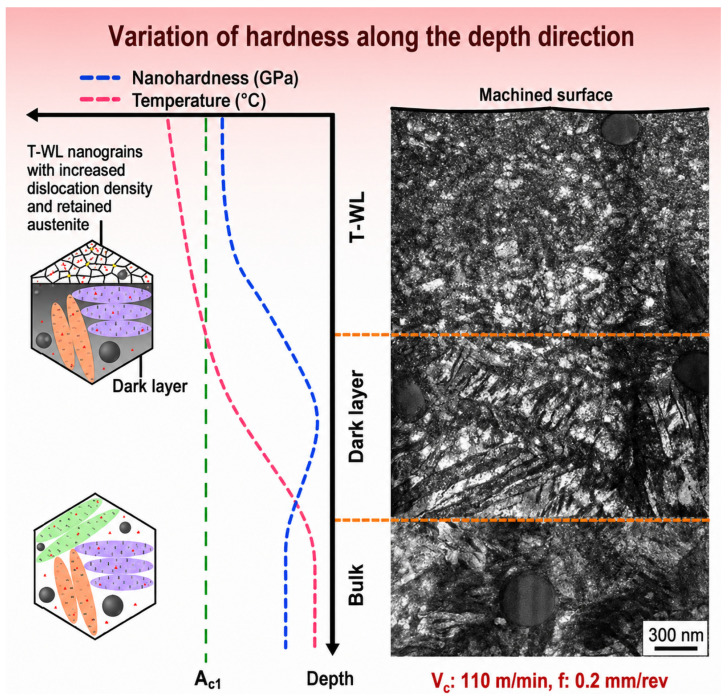
Depth-dependent nanohardness and temperature gradient profiles of the ground surface layer, with TEM microstructure and sublayer structure schematics, illustrating the hardness differences and micromechanisms among the T-WL hardened layer, tempered dark layer, and base material [102].

The core objective of mechanical property characterization of the metamorphic layer is to translate the previously described metallographic sublayer architecture into continuous depth profiles of mechanical parameters such as hardness, yield strength, and elastic modulus. Cross-sectional microhardness gradient testing is the most direct approach. By performing Vickers indentation at successive points along the depth direction on a polished and etched cross-section, a continuous hardness profile from the outermost surface to the base material can be obtained, delineating the boundaries and property differences of each sublayer through mechanical metrics. In the ground metamorphic layer of hardened GCr15 steel, the white layer hardness increases from approximately 650 HV in the base material to approximately 1000 HV, while the dark layer undergoes softening due to over-tempering, with hardness dropping below 500 HV across a softened zone approximately 50 μm wide. With further depth, the hardness gradually recovers to base material levels, and the full profile clearly demarcates the mechanical stratification of the white layer, dark layer, and bulk substrate [96]. A separate study reports comparable white layer hardness values in the range of 880–1020 HV for ground 52100 steel, with the dark layer slightly softer than the base material; moreover, the hardness profile correlates well with the retained austenite distribution and microstructural gradient [13]. The high hardness of the white layer originates from composite strengthening by cryptocrystalline/ultrafine martensite combined with a high dislocation density, whereas the dark layer softening reflects carbon depletion and dislocation recovery resulting from over-tempering. Figure 13 illustrates the depth-dependent nanohardness and temperature gradient evolution in the ground surface layer, accompanied by TEM microstructure and sublayer structure schematics, explaining the hardness differences and underlying micromechanisms among the mechanically induced white layer (T-WL), the tempered dark layer, and the base material. The TEM image on the right reveals an ultrafine-grained T-WL region beneath the machined surface, underlain by a tempered dark layer. In the hardness curve on the left, the surface nanohardness is significantly higher than that of the base material, corresponding to the hardened white layer composed of high-dislocation-density nanocrystals and retained austenite. With increasing depth, once the grinding temperature crosses the $A_{c1}$ threshold, the hardness enters the dark layer region and exhibits a pronounced trough, fully consistent with the over-tempering softening mechanism. Continuing toward the base material, the temperature subsides and the hardness gradually recovers to the steady-state level of the bulk.

Owing to the load constraints of conventional micro-indentation, traditional Vickers testing struggles to obtain a sufficient number of valid data points within the white layer, which is typically only 10–20 μm thick. Consequently, nanoindentation has been introduced in recent years for mechanical characterization of the metamorphic layer, which offers ultra-low loads and high spatial resolution. Nanoindentation not only provides hardness and reduced elastic modulus but also enables the extraction of constitutive parameters such as yield strength and work hardening index through finite element inverse analysis of the complete loading–unloading load–displacement (P–h) curve. Liu systematically conducted nanoindentation tests and dimensional inversion analysis on the grinding metamorphic layer of 8Cr4Mo4V steel. The results show that the yield strength of the metamorphic layer is approximately 2427 MPa, representing an approximately 20% increase over the base material, confirming significant mechanical strengthening of the ground surface layer [18]. Combined with the four-sublayer structure established by Zhang et al. for the same material using crystal plasticity finite element modeling [19], the depth-dependent mechanical gradient nature of the metamorphic layer is well validated. In summary, microhardness profiling and nanoindentation inversion together constitute a complete mechanical characterization methodology spanning from macroscopic property evaluation to constitutive parameter determination.

### 4.4. Residual Stress Measurement of the Grinding Metamorphic Layer

**Figure 14:**
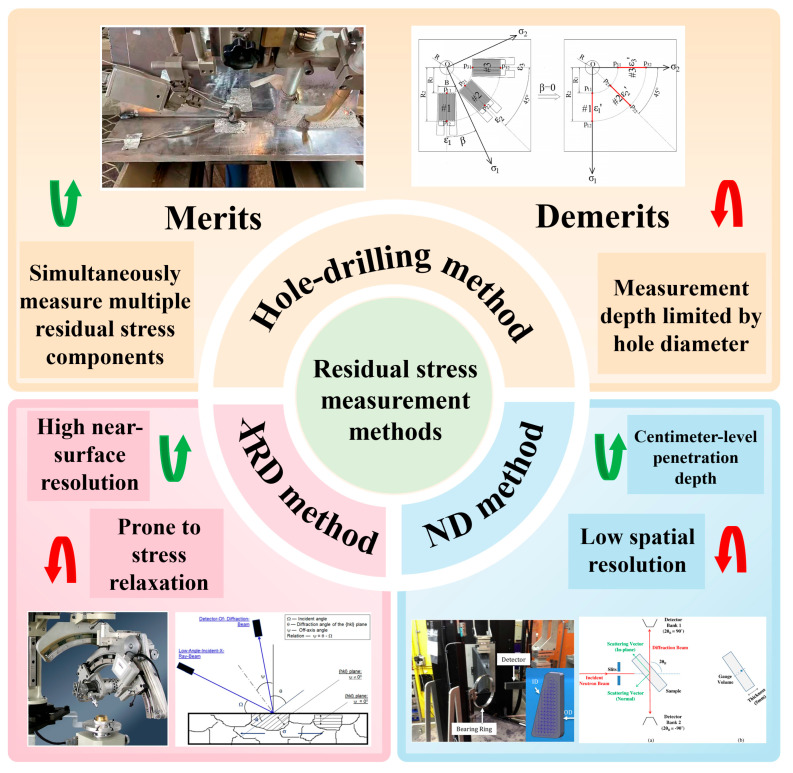
Schematic diagrams and comparative evaluation of three residual stress measurement techniques: the blind-hole method, X-ray diffraction, and neutron diffraction [106,107,108].

Residual stress is a critical mechanical parameter that links grinding process parameters to the service performance of bearings. Its depth distribution directly governs the influence of the metamorphic layer on fatigue, wear, and dimensional stability. The primary methods for obtaining residual stress profiles in the metamorphic layer are the X-ray diffraction (XRD) sin^2^ψ method, neutron diffraction, and the blind-hole method. These three techniques are complementary in terms of spatial resolution, penetration depth, and measurable stress components. Figure 14 systematically summarizes the core advantages and limitations of each technique. The XRD sin^2^ψ method is the most mature and widely used technique for surface residual stress measurement. Combined with electrolytic polishing for layer-by-layer material removal, it can acquire continuous residual stress depth profiles over tens to hundreds of micrometers [103,104]. Its limitation lies in the time-consuming nature of layer-by-layer removal and the potential for stress relaxation introduced during the removal process, which requires correction. Neutron diffraction, by virtue of the centimeter-scale penetration depth of neutrons through steel, enables non-destructive two-dimensional imaging of residual stress across entire bearing ring cross-sections [23]. However, its spatial resolution is limited by the millimeter-scale neutron beam size, so subsurface details are typically complemented by XRD. The blind-hole method measures the strain released by drilling a small hole using a strain rosette attached to the surface, then inverts the residual stress profile through integral methods. Its advantage lies in the ability to simultaneously obtain multi-directional stress components, but the measurement depth is limited by the hole diameter and the technique is operationally demanding [105].

The typical residual stress profile of the grinding metamorphic layer exhibits a zonal character, the morphology of which depends on the relative contributions of grinding heat and grinding force in the contact zone, as well as the material’s heat treatment state. In ground GCr15 steel, XRD measurements reveal that the subsurface region typically exhibits tensile residual stress on the order of approximately +300 to +400 MPa, which increases with grinding depth. Beneath this tensile zone, a compressive stress region develops, and at greater depths the stress gradually decays to near-zero levels approaching the base material [13,96]. This multi-zone structure of surface tension, subsurface compression, and deep decay directly reflects the competition between grinding heat and grinding force. Under high heat input conditions such as dry grinding or when using wheels with poor thermal conductivity, thermal effects dominate, significantly enhancing the surface tensile stress. In contrast, when cubic boron nitride wheels are used, their superior thermal conductivity carries away a large portion of the grinding heat, allowing the surface compressive residual stress to be maintained at approximately −600 to −1100 MPa [109].

It is worth emphasizing that residual stress is not an isolated consequence of a single grinding operation, but a cumulative response to the entire manufacturing route of bearing rings. Neutron diffraction investigations on 52100 bearing rings have shown that each processing step introduces a distinct residual-stress signature from the outer-diameter surface toward the core [102]. After soft cutting or rough machining, tensile residual stress is mainly confined to a very shallow surface region, whereas the subsurface and core retain comparatively lower stress levels. Subsequent heat treatment markedly reduces the surface tensile stress and promotes the formation of a shallow compressive layer beneath the surface. After final hard turning or finishing, the combined effects of prior heat treatment and near-surface mechanical deformation generate a more pronounced compressive residual-stress layer, characterized by both higher compressive magnitude and greater penetration depth. In contrast, the stress state in the core is less affected by the final finishing step and largely preserves the residual-stress pattern established during heat treatment.

From a service-performance perspective, this through-depth stress evolution is significant because the magnitude, sign, and depth of residual stress jointly influence rolling contact fatigue resistance. In 52100 bearing rings, quantitative assessments combining XRD depth profiling with fatigue testing have indicated that higher levels of subsurface compressive residual stress can effectively improve the L10 fatigue life [107].

### 4.5. Non-Destructive Testing of Ground Surfaces

**Figure 15:**
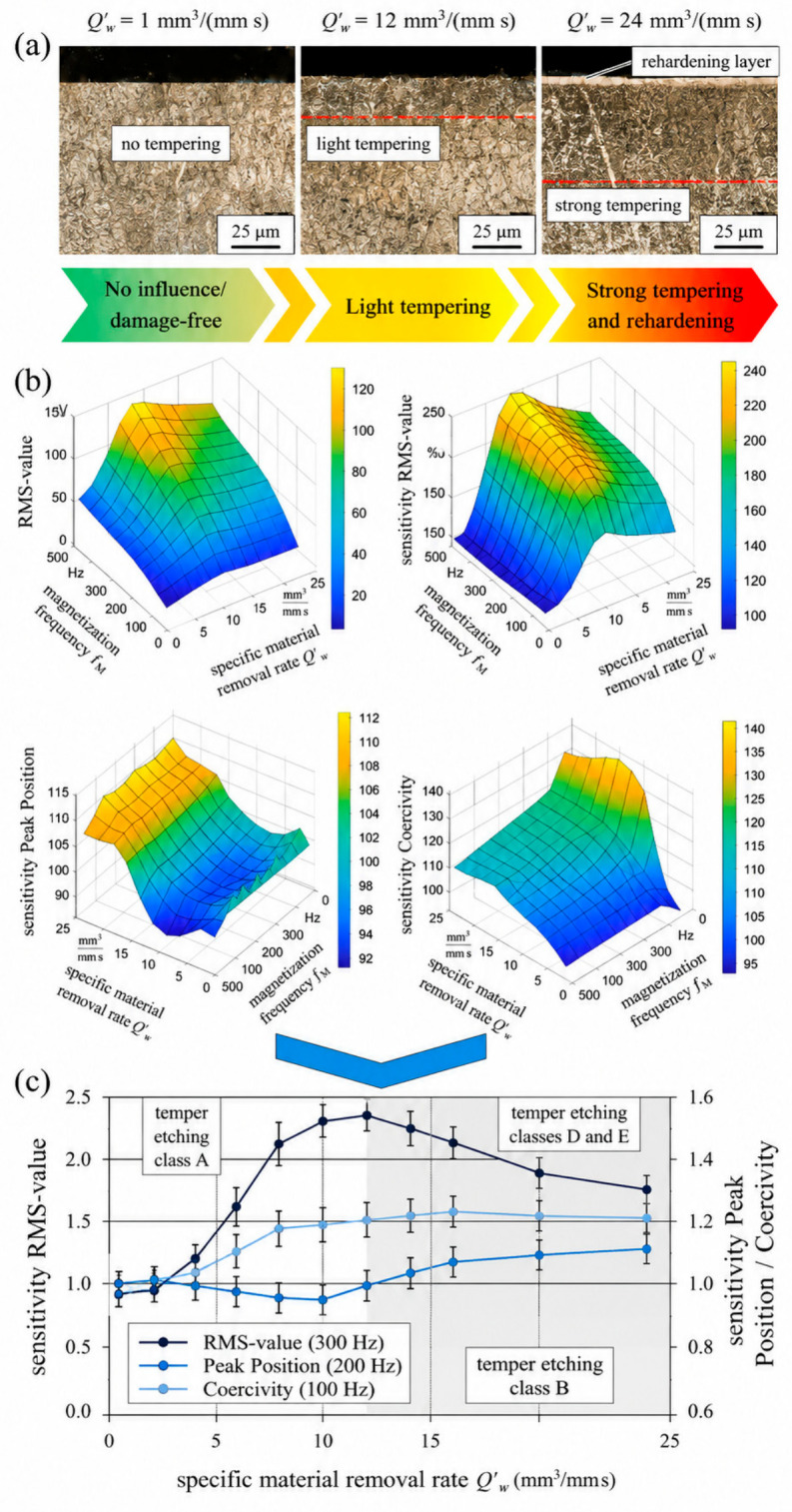
Multi-parameter BNA characterization for distinguishing tempering and re-hardening grinding burns [115]. (**a**) Metallographic morphology of the ground surface layer at different specific material removal rates Q′w; (**b**) three-dimensional response surface of BNA characteristic parameters under coupled magnetization frequency and material removal rate; (**c**) variation in BNA characteristic parameters with specific material removal rate at a representative magnetization frequency.

Grinding burn is among the most critical defects requiring immediate screening in bearing grinding operations, as it directly compromises surface integrity and, ultimately, service safety [98,110]. To meet the high-throughput demands of production lines, Barkhausen noise analysis (BNA) has emerged as the most representative non-destructive technique for detecting grinding-induced thermal damage [30,111]. Its principle is based on the electromagnetic noise signal generated by irreversible jumps of magnetic domain walls in ferromagnetic materials under an alternating magnetic field. Microstructural defects and hardness variations alter the mobility of domain walls, thereby changing the BNA amplitude and envelope curve shape [21,112]. In response surface analysis of cylindrical grinding of 52100 bearing rings, a BNA signal exceeding approximately 90 mp corresponds to severe tempering softening and tensile residual stress [21]. However, the BNA response to thermal damage is non-monotonic. As the grinding temperature increases, the BNA signal first rises due to tempering softening; when the temperature exceeds approximately 275 °C and re-hardening occurs, the BNA root-mean-square (RMS) value instead decreases [113]. This characteristic provides a basis for distinguishing between tempering-type and re-hardening-type burns and has already been implemented for online BNA monitoring during grinding under production conditions [114]. To improve diagnostic reliability, the BNA envelope peak position and coercivity are further used in combination to effectively differentiate between tempered and re-hardened states [115]. Figure 15a presents metallographic morphologies directly illustrating the evolution of grinding-induced thermal damage with increasing specific material removal rate. Figure 15b shows a three-dimensional response surface, demonstrating that the BNA characteristic parameters exhibit a non-monotonic response to grinding heat input, and that the magnetization frequency significantly modulates signal sensitivity. Figure 15c quantifies this trend: the RMS signal rises continuously with increasing tempering severity, peaks at *Q′_w_* ≈ 10 mm^3^/(mm·s), and then decreases at higher heat inputs due to surface re-hardening. The peak position and coercivity follow distinctly different evolution trends. By combining these parameters with metallographic etching classification standards, different thermal damage regimes can be delineated, confirming that joint multi-parameter BNA can effectively distinguish among mild tempering, severe tempering, and surface re-hardening burns, thereby improving the reliability of damage assessment.

Compared with the conventional nital etching inspection method, BNA offers a significant advantage in detection sensitivity for mild grinding burns. In the online grinding monitoring of a carburized raceway for a Φ1650 mm wind turbine main bearing, when the surface exhibited only mild tempering that was nearly indistinguishable by nital etching, the magnetic Barkhausen noise signal had already shown a clear decrease [31]. Beyond detecting the presence of burns, the position of the first MBN envelope peak can independently and linearly track the effective case depth of induction-hardened layers (±200 μm), independent of the burn state [111], enabling simultaneous non-destructive evaluation of both case depth and burn condition. Furthermore, in grinding experiments on hardened 52100 bearing steel, BNA parameters exhibit a good linear correlation with residual stress measured by XRD [112]. Acoustic emission (AE) technology provides an alternative route for online grinding burn monitoring. AE signals are highly sensitive to changes in grinding conditions; when grinding burn occurs, both the instantaneous energy of the AE signal and the second-order central moment of its spectrum decrease significantly [116]. Further studies have correlated the characteristic frequency and amplitude of AE signals with wheel condition and burn initiation [117], and have explored the potential of high-frequency domain analysis and cross-wavelet time–frequency methods for real-time AE-based burn detection [118].

Building on the established characterization techniques discussed above, several emerging methods offer potential to address remaining gaps in probing the grinding-affected layer at higher resolution and in greater depth. Synchrotron radiation diffraction and high-energy X-ray diffraction (HE-XRD) could provide non-destructive depth-resolved phase and residual stress information across the affected layer in a single measurement, although their systematic application to grinding-induced surface layers in bearing steels has not yet been reported. Three-dimensional EBSD (3D EBSD), combining serial sectioning with EBSD mapping, could reveal the three-dimensional morphology of the white layer and the spatial connectivity of grain refinement, though the method remains time-consuming and has not been applied in this specific context. Atom probe tomography (APT) offers near-atomic-scale compositional mapping capable of quantifying carbon supersaturation and carbide dissolution at the nanoscale within the white layer, but its extremely small sampling volume poses challenges for capturing representative gradient features. The application of these emerging techniques to the specific problem of grinding-affected layer characterization in bearing steels represents a promising direction for future investigation.

## 5. Influence of Grinding Process Parameters on Metamorphic Layer Structure and Residual Stress

The following subsections review the influence of individual grinding parameters on metamorphic layer structure and residual stress. It should be noted that the results cited in this section were obtained under a range of different experimental conditions, including varying steel grades, wheel types, cooling methods, and parameter ranges. The reported sensitivities and thresholds are therefore condition-dependent. The relative importance of each parameter may shift with steel grade. For example, the lower carbide thermal stability in AISI 52100 amplifies the influence of depth of cut relative to M50. Wheel thermal conductivity also plays a role: CBN suppresses thermal effects while Al_2_O_3_ amplifies them. Cooling effectiveness and the measured output metric, whether white layer thickness, dark layer thickness, residual stress, or hardness, further influence the observed sensitivity ranking. Readers should interpret individual findings within their specific experimental context rather than as universally applicable laws.

### 5.1. Influence of Depth of Grinding

Increasing the depth of cut simultaneously elevates both mechanical force and heat input, but the two do not increase symmetrically. The thermal input grows far more rapidly than the mechanical input, making depth of cut an effective parameter for tuning the thermal–mechanical competition [119]. Gong summarized this as a dynamic competition among three mechanisms [48]. Mechanical stress originates from non-uniform plastic deformation caused by abrasive grain plowing and tends to generate compressive residual stress at the surface. Thermal stress arises from the temperature gradient between the surface and subsurface and tends to produce tensile residual stress. Phase transformation stress depends on the relative magnitudes of volumetric expansion during martensitic transformation and contraction during tempering. Varying the depth of cut essentially alters the relative weights of these three stress contributions.

In precision grinding of AISI 52100 bearing steel, a critical depth of cut was identified by fixing the wheel speed and workpiece speed while varying only the depth of cut. At a depth of 20 μm, the peak grinding zone temperature was only 606 °C, and the metamorphic layer was dominated by mechanical effects. Severe plastic deformation formed subgrain boundaries through DRV, with subgrain rotation refining grains to the nanoscale. The resulting white layer was extremely thin and discontinuous, and no dark layer formed beneath it because the temperature remained below the tempering threshold. When the depth of cut increased to 25 μm, the peak temperature jumped to 778 °C, just exceeding $A_{c1}$ and triggering austenitization. At a depth of 40 μm, the temperature further rose to 1109 °C, original carbides dissolved within the extremely short contact time, and carbon and alloying elements re-dissolved into the austenite. Subsequent rapid self-quenching drove martensitic transformation, forming a carbon-supersaturated nanocrystalline white layer approximately 30 μm thick, beneath which a dark layer developed as the temperature fell between the tempering range and $A_{c1}$. Szkodo observed a similar trend in single-pass grinding of medium-carbon steel, where increasing depth of cut significantly raised both the carbon supersaturation and dislocation density in the white layer [120]. Hou et al. further found in internal grinding of hardened GCr15 that increasing the depth of cut from 0.1 mm to 0.3 mm increased the hardened penetration depth (HPD) from approximately 0.4 mm to 0.92 mm [121]. The variable-depth grinding experiments of Zhang et al. confirmed that the depth distribution profile of the hardened layer can be actively controlled by varying the depth of cut along the grinding path [122].

**Figure 16:**
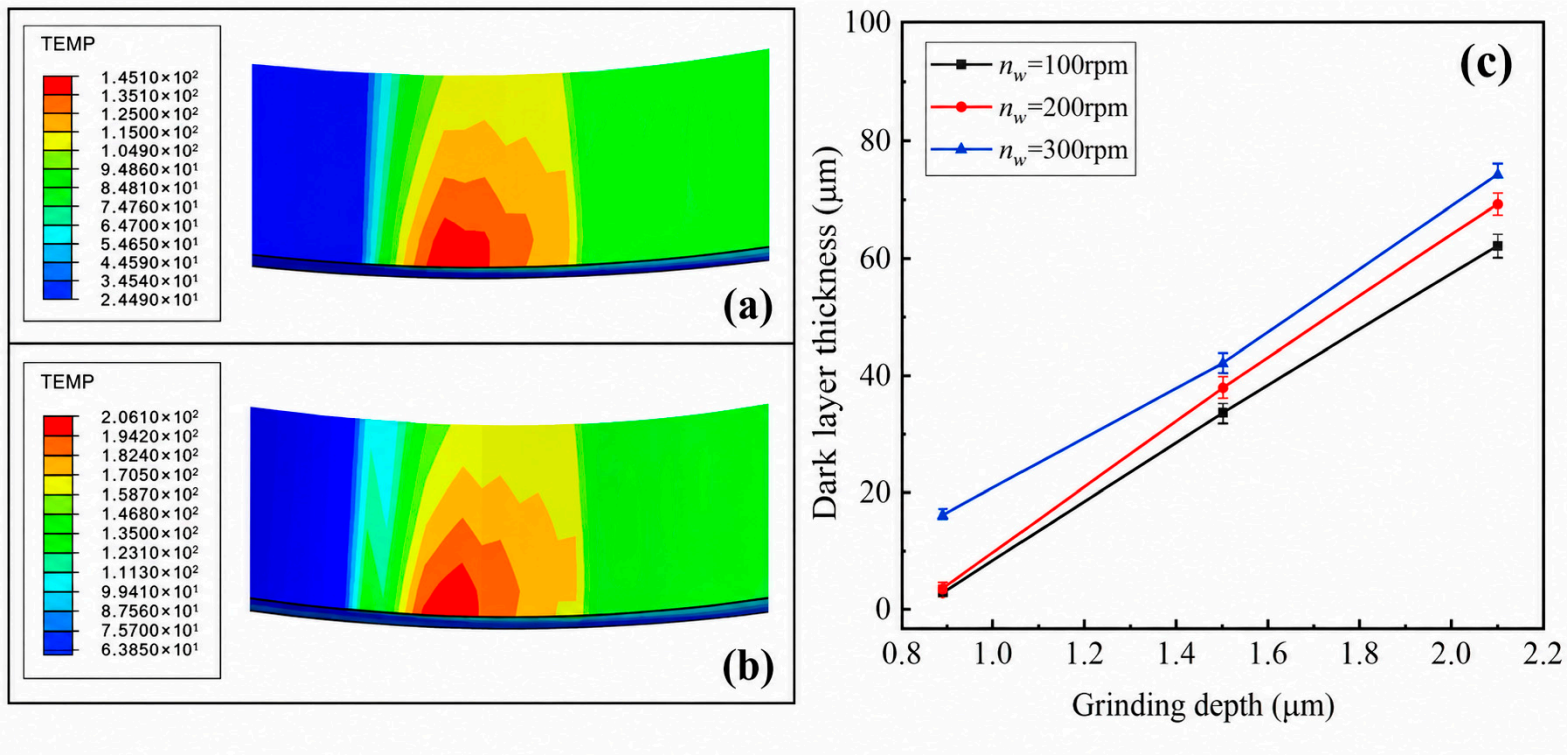
Temperature field distribution contours at different depths of cut (wheel speed vs = 40 m·s^−1^, workpiece speed nw = 100 rpm) and the influence of grinding depth on dark layer thickness [48]. (**a**) Depth of cut ae = 0.9 μm; (**b**) depth of cut ae = 1.5 μm; (**c**) effect of grinding depth on dark layer thickness at different workpiece speeds.

In a study of conical roller bearing raceway grinding, the influence of depth of cut on the grinding temperature field and tempered dark layer thickness was analyzed [48]. Figure 16a,b present finite element simulation results of the temperature field at two depths of cut (ae = 0.9 μm and ae = 1.5 μm) under identical wheel speed (vs = 40 m·s^−1^) and workpiece speed (nw = 100 rpm). When the depth of cut increased from 0.9 μm to 1.5 μm, the maximum grinding temperature rose from 145.1 °C to 206.1 °C. This increase in depth of cut simultaneously increased the maximum undeformed chip thickness and the abrasive grain penetration depth, increasing the number of grains engaged in plowing and cutting, raising the normal grinding force, and ultimately causing a sharp temperature rise in the grinding arc zone. Figure 16c shows the effect of grinding depth on dark layer thickness, which increases approximately linearly with depth of cut. This trend is consistent with the temperature field evolution, as the increase in maximum undeformed chip thickness and normal grinding force leads to rapidly rising temperatures in the grinding arc zone and consequently accelerated dark layer growth.

The influence of depth of cut on residual stress is equally significant. Through combined AISI 52100 grinding experiments and multiphysics FEM simulations at *v*_w_ = 12 m/min and *a*_e_ = 0.2 mm, Rajaei found that the re-hardened zone within approximately 40 μm of the surface exhibited compressive residual stress, while the underlying tempering zone at depths of approximately 40–300 μm showed tensile residual stress [82]. Similarly, Wang’s integrated modeling of bearing ring raceway grinding further elucidated the coupling pathways among mechanical stress, thermal stress, and phase transformation stress, noting that even when the temperature remains below the phase transformation threshold, heat flow can still weaken the compressive residual stress [16]. The study by Deng also confirmed the significant modulating effect of grinding parameters on surface residual stress [123].

### 5.2. Influence of Feed Rate

**Figure 17:**
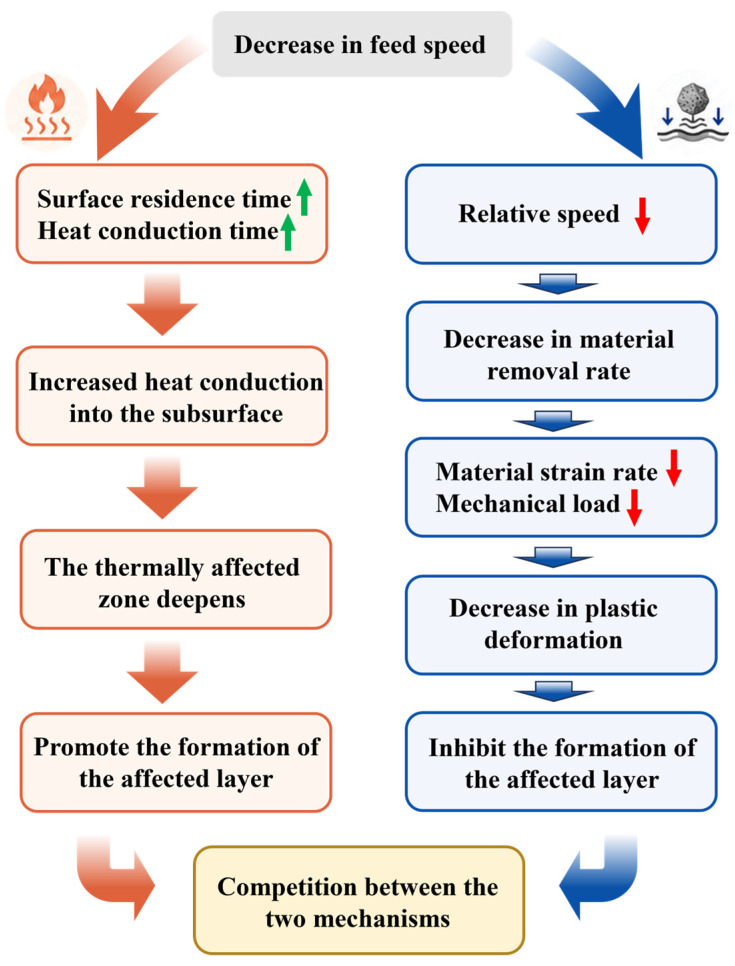
Schematic diagram of the evolution logic of two competing mechanisms when the feed rate is reduced.

The feed rate simultaneously influences two factors: the dwell time of grinding heat on the surface and the material strain rate. These two factors act on the metamorphic layer structure in opposing directions, and the net effect depends on which factor dominates within a given parameter window. Figure 17 illustrates the evolution logic of these two competing mechanisms. A lower feed rate prolongs heat conduction time, favoring deeper penetration of the heat-affected zone. However, it also reduces the relative velocity between the wheel and the workpiece, decreasing the material removal per abrasive grain and thereby weakening the force input. This two-way competition means that the influence of feed rate on the metamorphic layer is less straightforward and monotonic than that of depth of cut or wheel speed.

**Figure 18:**
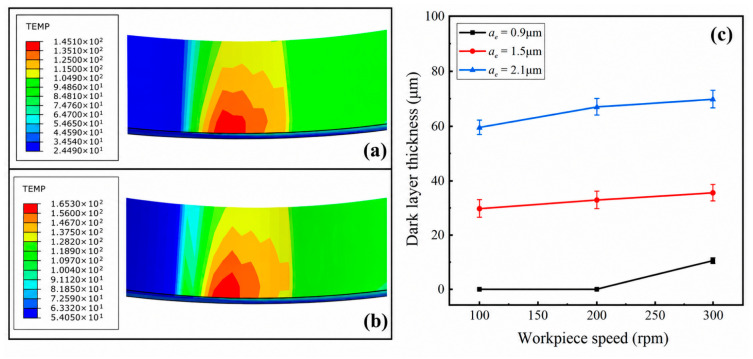
Temperature field distribution contours at different workpiece speeds (wheel speed vs = 40 m·s^−1^, depth of cut ae = 0.9 μm) and the effect of workpiece speed on dark layer thickness [48]. (**a**) Workpiece speed nw = 100 rpm; (**b**) workpiece speed nw = 200 rpm; (**c**) effect of workpiece speed on dark layer thickness at different depths of cut.

In rail grinding experiments across a feed rate range of 600–2400 mm/min, Uhlmann demonstrated that the feed rate is a key parameter affecting surface roughness and hardness. However, the hardness variation was not controlled by thermal softening. At the lowest feed rate (600 mm/min), the contact time was longest and work hardening strongest, causing the surface hardness to actually increase [124]. The hardness peak occurred at an intermediate feed rate and then decreased with further increases in feed rate which is driven by mechanical work hardening rather than thermal accumulation-induced softening. In the grinding of bearing components, the feed rate and the workpiece rotational speed jointly determine the equivalent depth of cut. In conical roller bearing outer ring inner raceway grinding, increasing the workpiece speed was confirmed to raise the grinding temperature and increase the dark layer thickness [48]. Figure 18a,b present finite element simulation results of the temperature field under two workpiece speeds (nw = 100 rpm and nw = 200 rpm) at a constant wheel speed of vs = 40 m·s^−1^ and depth of cut ae = 0.9 μm. When the workpiece speed increased from 100 rpm to 200 rpm, the maximum temperature rose from 145.1 °C to 165.3 °C. This increase in workpiece speed raised the maximum undeformed chip thickness in the grinding arc zone, the grinding force, and the heat flux density, ultimately elevating the grinding arc temperature. Figure 18c shows the effect of workpiece speed on dark layer thickness, which increases slowly with increasing workpiece speed, reflecting the modest temperature rise observed in Figure 18a,b. The increase in workpiece speed similarly increases the maximum undeformed chip thickness, thereby intensifying grinding heat and raising the grinding arc temperature. However, because a higher workpiece speed shortens the grinding duration for each surface element of the raceway, the time available for heat accumulation is reduced, and the grinding arc is immediately cooled by the grinding fluid after passage. Consequently, the dark layer thickness increases only gradually.

With respect to residual stress, the feed rate alters the relative weighting of thermal exposure time and strain rate, directly determining whether the surface stress state is dominated by thermally induced tensile stress or mechanically induced compressive stress. Rajaei compared residual stress under different workpiece speeds in a fully coupled thermal–mechanical–metallurgical FEM of AISI 52100 grinding. At low workpiece speeds, the grinding heat dwell time at the surface was longer, increasing the penetration depth of thermally induced tensile stress and significantly extending the depth range over which tensile stress prevailed [82]. At high workpiece speeds, the heat conduction time was shortened, the heat-affected zone became shallower, and mechanical effects were relatively enhanced. Compared with depth of cut and wheel speed, the primary role of feed rate lies in controlling the duration of thermal exposure.

### 5.3. Influence of Wheel Speed

The effect of wheel speed on the metamorphic layer is twofold. An increase in wheel speed reduces the maximum undeformed chip thickness per abrasive grain, decreasing the grinding force and mitigating mechanical input. Simultaneously, however, the number of active cutting grains per unit time increases sharply, intensifying frictional heating and substantially raising the heat flux density. In bearing steel grinding, wheel speed thus becomes the primary determinant of metamorphic layer thickness and residual stress.

Huang investigated the CBN high-speed grinding of hardened steels over a wheel speed range of 60–150 m/s [62]. The white layer thickness increased monotonically with wheel speed, with heating rates reaching 10^5^–10^6^ °C/s and cooling rates exceeding 10^4^ °C/s. The peak surface temperature exceeded $A_{c1}$ under all conditions. The retained austenite content recovered from approximately 4.5% in the base material to 19.1% and 32.5%, and the martensite subgrain size was refined from approximately 86.7 nm in the base material to 6.2–10.1 nm within the white layer. These findings indicate that the white layer is dominated by thermally induced secondary quenching transformation, with negligible plastic deformation contribution at high speeds.

In bearing ring grinding, Cheng found that increasing the wheel speed raises the grinding zone temperature and correspondingly increases the dark layer thickness. Moreover, the non-uniform distribution of the temperature field along the raceway wall thickness causes the dark layer to be thicker at the thin-walled end [48]. Multi-factor experiments established the following hierarchy of influence on metamorphic layer thickness: wheel speed > depth of cut > feed rate [125]. Shu revealed the mechanism directly related to the speed effect through single-grain grinding studies [126]. Under dry grinding conditions, the peak temperature rise of sharp ceramic Al_2_O_3_ grains remained below the transformation temperature of gear steel; however, after grain wear, the peak temperature rise could exceed the transformation temperature.

In terms of residual stress, increasing the wheel speed raises the heat flux density, enhancing the tensile contribution of thermal stress and shifting the surface stress toward tension. Using rolling and laser heating as analog processes to separate mechanical and thermal effects before recombining them, Duscha established a qualitative model of residual stress in CBN speed-stroke grinding of 100Cr6 steel and validated it through grinding experiments [127]. At low speeds, thermal effects dominated, with surface tensile residual stress exceeding 500 MPa. At high speeds, mechanical impact dominated, producing compressive residual stress. In high-speed grinding of 52100 steel, the measured residual stresses in both the grinding direction and the perpendicular direction were tensile, reaching 950–1600 MPa under high-speed conditions, and were strongly correlated with white layer depth. This further indicates that when the speed is sufficiently high, the dynamic competition between thermally induced stress and the compressive phase transformation stress generated by martensitic transformation in the white layer is ultimately resolved in favor of thermal effects.

### 5.4. Influence of Wheel Characteristics

Wheel characteristics influence the heat partition ratio between the workpiece and the wheel through the thermal conductivity of the abrasive grains, the cutting geometry of the grains, and the self-sharpening ability of the wheel, deciding the grinding force and heat generation rate [128].

Differences in abrasive thermal conductivity are directly reflected in the thickness and uniformity of the white and dark layers. Ji compared the dry grinding of hardened 1045 steel using brazed CBN wheels and conventional SiC wheels [129]. Within the same depth of cut range, the CBN wheel consistently produced lower surface roughness than the SiC wheel, maintained subsurface hardness above the base material level, and sustained compressive residual stress throughout. In contrast, the SiC wheel exhibited surface burn, tearing, and microcracks once the depth of cut exceeded 0.05 mm, with the residual stress transitioning from compressive to tensile. The fundamental difference between the two abrasives lies in their heat dissipation pathways: the aluminum matrix of the CBN wheel conducts grinding heat away from the contact zone, whereas the poor thermal conductivity of the SiC wheel allows heat to accumulate in the workpiece surface layer, causing thermal softening to override work hardening and leading to a sharp deterioration of surface integrity.

Wang further demonstrated the critical importance of grain self-sharpening in gear grinding of 20CrMnTi steel [130]. Microcrystalline alumina (MA) wheels, whose grains continuously expose fresh cutting edges through microfracture, reduced normal and tangential grinding forces by approximately 30–40% compared with white alumina (WA) wheels. The metamorphic layer thickness decreased from approximately 55 μm to approximately 40 μm with reduced thickness fluctuation, the incidence of grinding burn was significantly lowered, and the surface compressive residual stress increased from approximately −250 to −300 MPa to approximately −350 to −400 MPa. The cutting-edge radius (40–105 μm) is the most important parameter affecting surface roughness, residual stress, white layer thickness, and subsurface hardness. A larger cutting-edge radius increased the maximum compressive residual stress from −570 to −1050 MPa but also increased the white layer thickness from zero to over 5 μm and worsened surface roughness [82].

Regarding residual stress in the grinding metamorphic layer, wheels with high thermal conductivity or sharp cutting edges, such as CBN and microcrystalline alumina, suppress thermal effects and favor mechanical dominance, producing higher and deeper compressive residual stress. Conversely, conventional Al_2_O_3_ or SiC wheels, with poor thermal conductivity or blunted cutting edges, allow thermal effects to accumulate, weakening compressive stress or even converting it to tensile stress. These differences are not isolated. Jouini demonstrated the influence of process type on surface integrity and service performance through a comparison of different finishing processes on AISI 52100 bearing rings [103]. Precision hard turning produced a hook-shaped subsurface compressive residual stress profile with a peak of approximately −680 MPa and a rolling contact fatigue life of 5.2 × 10^6^ cycles, approximately four times the 1.2 × 10^6^ cycles obtained by conventional grinding. Adding a honing process after grinding to remove the white layer and improve surface roughness restored the fatigue life to 3.2 × 10^6^ cycles.

### 5.5. Influence of Cooling Conditions

Cooling conditions allow independent control of the heat dissipation rate without altering the grinding parameters themselves, enabling a transition in the governing mechanism of metamorphic layer formation [131]. This mechanistic transition was demonstrated in supercritical CO_2_ cryogenic grinding (SCGT) of 300M ultra-high-strength steel [67]. Using a zirconia-alumina wheel at a speed of 20 m/s, a depth-decreasing gradient strain field was generated. The high cooling rate of supercritical CO_2_ effectively suppressed grinding-heat-induced martensite-to-austenite transformation, leaving the surface microstructural evolution entirely dominated by dislocation slip. This produced a gradient microstructure comprising a nanocrystalline surface layer, ultrafine grains, ultrafine lamellae, dislocation structures, and the base material, without the formation of a phase-transformation-driven white layer. This gradient structure corresponded to a microhardness decrease from 7.8 GPa at the surface to 5.8 GPa at a depth of 100 μm.

He developed a cellular automaton model for martensitic transformation on the ground surface [84], revealing the influence of peak grinding temperature and cooling rate on the final martensite grain size. Lower grinding temperatures and higher cooling rates produced finer martensite, which in turn led to higher hardness and greater wear resistance on the ground surface.

The influence of cooling conditions on the grinding metamorphic layer extends beyond temperature control to include indirect modulation of the friction contact state through the chemical properties of the cooling medium. In the context of cryogenic cooling, Reddy and Ghosh compared the grinding performance of Al_2_O_3_ wheels for AISI 52100 bearing steel under liquid nitrogen (LN_2_), dry grinding, and soluble oil [132]. LN_2_ cooling increased the wheel G-ratio from 19 (dry grinding) to 48 (an approximately 280% improvement). Spherically melted chips were significantly less abundant in the LN_2_ condition than in dry grinding or soluble oil environments, indicating that the cryogenic medium effectively suppressed thermal damage in the grinding zone. However, LN_2_ cooling also nearly doubled the spindle power consumption compared with dry grinding, accompanied by side effects such as prolonged spark-out time and reduced dimensional accuracy.

It is important to recognize that the five parameters discussed above do not act independently in practice; their effects are coupled through the shared thermal–mechanical framework. For example, the critical depth of cut for the mechanical-to-thermal transition is not a fixed value but varies with wheel speed: at higher wheel speeds, the increased heat flux density lowers the depth of cut at which the transition occurs [60,118], while CBN wheels—through their higher thermal conductivity—can raise this threshold by effectively dissipating heat from the contact zone [128]. Similarly, effective cooling can suppress thermal effects even at high depths of cut and wheel speeds, effectively raising the threshold for thermal dominance [131]. Feed rate interacts with both depth of cut and wheel speed through the undeformed chip thickness per grain, which determines the force per grain and the local heat generation rate. These coupling effects mean that the optimal combination of parameters for controlling the affected layer cannot be determined by optimizing each parameter in isolation; rather, it requires a system-level approach that accounts for the specific steel grade, wheel type, and cooling configuration.

## 6. Summary and Outlook

### 6.1. Conclusions

Research on grinding-induced metamorphic layers has progressed from isolated single-factor studies toward a systematic framework integrating thermo-mechanical-phase-transformation coupling, gradient microstructures, and service performance. The main conclusions of this review are summarized as follows:

- The grinding metamorphic layer in bearing steels forms via thermal–mechanical–phase-transformation coupling and has a three-layer gradient structure. Its dominant mechanism shifts from plastic deformation to thermal phase transformation once the surface temperature exceeds $A_{c1}$, causing a sharp increase in white layer thickness and dark layer softening.
- Carbide thermal stability determines the layer morphology. Low-alloy bearing steels form well-defined white layers and dark layers, while high-alloy steels show a gentle gradient without sharp boundaries due to partially dissolved stable MC carbides.
- A multi-scale complementary characterization system is established, covering microstructure analysis, mechanical property profiling, residual stress measurement and non-destructive grinding burn detection.
- Five key grinding parameters collectively regulate the structure and residual stress of the affected layer through the thermal–mechanical competition framework. The influence of each parameter follows a hierarchical order, but this ranking varies with steel grade, wheel type, and cooling conditions, and is therefore not fixed. These parameters are strongly coupled, requiring system-level optimization tailored to specific working conditions.

### 6.2. Future Opportunities

Research on the grinding metamorphic layer has evolved from single-factor investigations into a systematic framework encompassing thermal–mechanical–phase-transformation multi-field coupling, gradient microstructure, and service performance. Nevertheless, based on a survey of the literature over the past two decades, the following three directions still present significant research gaps.

- Limited integration of coupled models with bearing manufacturing processes.

Current grinding simulations for bearing steels after heat treatment can incorporate temperature evolution, phase transformation kinetics, transformation strain, and the TRIP effect, producing residual stress predictions consistent with XRD measurements. However, the initial residual stress inherited from heat treatment is often neglected. Moreover, increases in critical transformation temperature caused by the heating rate and decreases caused by contact stress have not been considered simultaneously. Therefore, a unified framework incorporating initial stress and changes in transformation temperature is required to support process selection throughout the complete bearing manufacturing chain.

- Incomplete understanding of process–structure–performance relationships.

Grinding depth and wheel speed strongly influence the metamorphic layer thickness and residual stress state, while white layer depth is closely associated with tensile residual stress. Although honing after grinding can remove the white layer and extend bearing life, the effects of the metamorphic layer on wear resistance and long-term dimensional stability remain poorly understood. In particular, quantitative data on metamorphic layer thickness, residual stress gradients, and retained austenite content are still lacking.

- Experimental and modeling studies of the grinding metamorphic layer on real bearing ring raceways are lacking.

Most existing studies are based on simplified planar specimens rather than actual bearing raceways. Investigations of the outer raceway of the TR0708 tapered roller bearing have revealed pronounced spatial variations in grinding temperature and metamorphic layer thickness, as well as discontinuous white layer formation caused by the random engagement of abrasive grains. These characteristics cannot be adequately reproduced by conventional surface grinding experiments. Future studies should therefore extend existing models from planar specimens to curved raceway surfaces and from components with uniform thickness to those with spatially varying thickness.

## Data Availability

No new data were created or analyzed in this study. Data sharing is not applicable to this article.
